# Host Evolutionary History Drives Prokaryotic Diversity in the Globally Distributed Sponge Family Petrosiidae

**DOI:** 10.1111/mec.70186

**Published:** 2025-11-28

**Authors:** N. van der Windt, B. Paix, J. C. Biesmeijer, R. Ambo‐Rappe, Y. M. Huang, K. G. S. Nirbadha, D. Sipkema, N. J. de Voogd

**Affiliations:** ^1^ Naturalis Biodiversity Center Leiden The Netherlands; ^2^ Institute of Environmental Sciences (CML) Leiden University Leiden The Netherlands; ^3^ UMR CARRTEL INRAE—Université Savoie Mont‐Blanc Thonon‐les‐Bains France; ^4^ Faculty of Marine Science and Fisheries, Department of Marine Science Hasanuddin University Makassar Indonesia; ^5^ National Penghu University of Science and Technology Magong Taiwan; ^6^ National Aquatic Resources Research and Development Agency (NARA) Colombo Sri Lanka; ^7^ Laboratory of Microbiology Wageningen University Wageningen The Netherlands; ^8^ Institute of Biology (IBL) Leiden University Leiden The Netherlands

**Keywords:** Haplosclerida, microbial drivers, microbiome, phylogenetic signal, phylosymbiosis, porifera

## Abstract

Sponge microbial communities play a crucial role in marine ecosystem functioning and serve as a rich source of bioactive compounds. While host identity is recognised as a major determinant of microbiome diversity, the underlying evolutionary mechanisms remain poorly understood. This study aimed to comprehensively assess phylosymbiosis patterns within the sponge family Petrosiidae. In total 21 sponge species, collected across a broad geographic scale, were examined to investigate how host phylogeny influences microbiome composition. Using 28S rRNA, 18S rRNA and COI gene barcoding to identify host sponges, combined with 16S rRNA gene amplicon sequencing to characterise prokaryotic communities, we provide evidence of phylosymbiosis through multiple analytical approaches, including distance‐based metrics and topological congruence. Our results show that host phylogeny and identity play a significant role in structuring sponge microbiomes, even at finer taxonomic resolutions. However, we observed notable incongruencies, where closely related sponge species exhibit divergent microbial communities that appear to be associated with depth or geographical location. This study represents the first large‐scale investigation of phylosymbiosis in sponges at the family level, providing valuable insights into the evolutionary and ecological drivers shaping sponge microbiomes, particularly in the sponge family Petrosiidae.

## 1 Introduction

Marine sponges have a long‐standing symbiotic relationship with a vast diversity of microbial organisms. Together, sponges and their microbial communities are vital components of marine ecosystems, contributing significantly to functional roles such as water filtration and nutrient cycling (Bell 2008). Furthermore, sponge microbiomes have been the focus of extensive research and exploration and represent a significant and promising source of bioactive compounds with potential applications in various fields (Sipkema 2017; Varijakzhan et al. 2021). The immense diversity of bioactive compounds found in sponges can be attributed to their rich microbial communities, which can comprise up to 35% of the sponges' biomass, highlighting their importance to the sponge host. Furthermore, the microbial diversity within sponges is exceptionally high, constituting one of the most intricate and complex host‐microbe associations in the marine environment (Thomas et al. 2016).

While the symbiosis between sponges and their microbes has been studied extensively (e.g., Díez‐Vives et al. 2020; Moitinho‐Silva et al. 2017; Robbins et al. 2021; Thomas et al. 2016), the drivers of microbial diversity are still not fully understood. Studies have shown that a key variable explaining sponge microbial diversity is host identity, with each sponge species harbouring unique microbial communities (Busch et al. 2022). Additionally, research into phylosymbiosis has demonstrated a strong link between sponge taxonomy and microbial communities. Phylosymbiosis is a concept where the microbial community composition is correlated to the host's evolutionary history. More specifically, greater host genetic distances are associated with increasingly distinct microbial community compositions (Brooks et al. 2016). While evolutionary processes may contribute to this pattern, phylosymbiosis is not solely an evolutionary concept (Lim and Bordenstein 2020). It may arise through several, potentially interacting, mechanisms such as codiversification or ecological filtering (Kohl 2020; Mazel et al. 2018). In sponges, phylosymbiosis has recently been investigated across multiple scales, including large‐scale studies at higher taxonomic levels (Thomas et al. 2016; Pankey et al. 2022), smaller studies with limited sample sizes (Easson and Thacker 2014; O'Brien et al. 2020; Schöttner et al. 2013), and on a cryptic species complex within the genus *Agelas* (Pankey et al. 2024). To the best of our knowledge, phylosymbiosis has not yet been comprehensively studied on a large scale at the sponge family level. To study phylosymbiosis in greater detail, we will analyze closely related species within a single clade, the sponge family Petrosiidae (order Haplosclerida, Class Demospongiae).

Petrosiidae sponges are globally distributed and inhabit a wide variety of marine ecosystems including temperate regions, coral reefs, the deep sea and mangrove habitats. While some species have a wide distribution range, for instance across the greater Caribbean basin, others are endemic to small geographic areas. All previously studied Petrosiidae are classified as high microbial abundance (HMA) sponges and house dense microbial consortia in their tissues (Cleary et al. 2024; Gloeckner et al. 2014; Moitinho‐Silva et al. 2017). Moreover, they are recognised as one of the most prolific sources of novel bioactive compounds among sponges (Leal et al. 2012; Lee et al. 2021). The current taxonomic classification of the Petrosiidae is based on morphological characteristics and at present 128 nominal species of Petrosiidae are accepted within four genera and two subgenera: *Acanthostrongylophora*, *Neopetrosia*, *Petrosia* (*Petrosia*), *Petrosia* (*Strongylophora*) and *Xestospongia* (de Voogd et al. 2025; Desqueyroux‐Faúndez and Valentine 2002; van Soest 1980). The combination of high species diversity and widespread occurrence makes Petrosiidae an ideal model for studying phylosymbiosis patterns.

Specifically, phylosymbiosis can be studied using multiple analytical approaches (Lim and Bordenstein 2020). The most commonly used methods to quantify phylosymbiosis include topological comparisons and distance‐based metrics. Topological comparisons are performed using the normalised Robinson‐Foulds (nRF) metric, which ranges from 0 (strong phylosymbiosis) to 1 (no phylosymbiosis). This metric quantifies the degree of topological similarity and assesses congruence between the host phylogenies and microbial dendrograms (Lim and Bordenstein 2020; Robinson and Foulds 1981). Tanglegrams provide a visual representation of this relationship, highlighting both congruences and incongruences. In addition, distance‐based metrics, such as the Mantel test (Mantel 1967), evaluate the correlation between host genetic distance and microbiome dissimilarities, testing whether microbial communities become increasingly distinct as host lineages diverge. In addition to phylosymbiosis, phylogenetic signal can be measured to investigate the extent to which a univariate trait, such as microbial richness, follows a phylogenetic structure and can be calculated using Pagel's Lambda (Pagel 1999). While all these metrics are employed in studies investigating phylosymbiosis, different methodologies and workflows are used, as there is no clearly established conceptual framework or workflow yet (Lim and Bordenstein 2020).

The objective of this study is to investigate evolutionary drivers of microbial diversity in Petrosiidae. More specifically, we aim to elucidate the extent to which prokaryotic diversity is explained by evolutionary lineages (phylogeny) of the host. Our research tests the hypothesis that phylosymbiosis patterns persist in closely related sponge species, where host phylogeny correlates with prokaryotic community structure. The significance of this study lies in the scale and resolution of our dataset. To the best of our knowledge, it represents the first systematic exploration of phylogenetic drivers of microbial diversity, including phylosymbiosis, at the sponge family level, which contributes valuable insights into the relationship between sponges and their microbiomes.

## 2 Materials and Methods

### 2.1 Sample Collection

Petrosiidae sponges have been collected from diverse locations and ecosystems in the Caribbean Region (71), Red Sea (3) and the Indo‐Pacific Region (95). These sampled habitats include marine caves, coral reefs, mangroves and the mesophotic zone. The sponges were collected using a submersible, SCUBA, dredging or snorkelling and subsequently preserved in 96% ethanol or RNA later for long‐term storage at −20°C. Samples were collected using SCUBA and snorkelling at depths shallower than 30 m, dredging was performed between 60 and 90 m depth, and sampling with the submersible was performed at depths between 48 and 189 m. Detailed information on all samples is summarised in Table S1.

### 2.2 DNA Extractions

For molecular analysis, pieces of tissue containing both the choanosome and ectosome of the sponges were subsampled. DNA extractions were performed using the MP Biomedicals FastDNA SPIN Kit for Soil with minor modifications to the manufacturer's protocol. Tissue lysis was carried out using a Qiagen Tissuelyser II, which involved a single bead beating cycle of 1.5 min at 30 Hz without the addition of the CD1 buffer, followed by two cycles of 5 min at 30 Hz with the addition of the CD1 buffer. The DNA was eluted in 100 μL of DES elution buffer and stored in the refrigerator until further processing.

### 2.3 Sponge Identification and Taxonomy

Sponges were identified by a combination of morphological characters and molecular characters to provide names or preliminary species groupings. Morphological characters used include classical characters such as growth form, skeletal characteristics and spicule characteristics.

For molecular identifications three common barcoding marker genes for sponges were used: 18S rRNA, 28S rRNA and cytochrome c oxidase subunit I (COI). The markers were amplified in a first PCR using the respective primer pairs SP18aF and SP18gR (Redmond et al. 2013), 28S‐C2‐fwd and 28S‐D2‐rev (Chombard et al. 1998) and dgLCO1490 and dgHCO2198 (Meyer et al. 2005) with nanopore sequence adaptors. More detailed information on PCR mixes and thermal cycler conditions is found in Table S2. In a bead clean‐up the PCR products were cleaned using a Cytena C.WASH machine before the second PCR to barcode each individual plate with 96 unique adaptors. After the second PCR, each individual plate was pooled by collecting 1 μL per sample using an OT2 Liquid Handler and each individual sub pool was purified using another bead clean‐up. Subsequently, end‐repair and dA‐tailing were performed on the sub pools using the NEBNext Ultra II End prep kit, followed by a 1.0× bead clean‐up to remove kit enzymes and buffer. DNA concentration in each pool was quantified using a Qubit flex and an equimolar mass of each sample was taken in a mixture of 7.5 μL for the next step. Native barcodes were ligated to each sub pool using the NEB Blunt/TA Ligase Master mix. Then sub pools were pooled in a single tube, followed by an AMPure XP 0.4× bead clean‐up. Finally native adapters were ligated using the NEBNext Quick ligation kit and a final quantification of the library was done on the Qubit Flex. The final library size was set up to 12 μL at 10–20 fmol. The library was sequenced on a MinION Flow Cell (R10.4.1).

Nanopore reads were assembled using NGSpeciesID 0.3.0 and medaka 1.8.0. Further analyses were performed in Geneious Prime. Sequences were blasted against the NCBI Core nucleotide database to identify sponge reads and were aligned with MAFFT (Katoh and Standley 2013). A maximum likelihood phylogenetic tree was generated using RAxML (Stamatakis 2014) using the GTR GAMMA I Nucleotide model and 1000 bootstrap replicates were generated using the Rapid Bootstrapping and search for the best‐scoring ML tree. All sequences were deposited in GenBank under the accession numbers PV350143–PV350311 (28S rRNA), PV351460–PV351603 (18S rRNA) and PV351187–PV351328 (COI) (Table S1).

### 2.4 16S rRNA Gene Amplicon Sequencing and Processing

Similar to the Nanopore protocol for sponge barcodes, for 16S rRNA gene amplicon sequencing of the sponge prokaryotic symbionts a library was prepared using a two‐step PCR protocol. The V4‐V5 region of the 16S rRNA gene was amplified using the primers 515F‐Y (GTGYCAGCMGCCGCGGTAA) (Parada et al. 2016) and 926R (CCGYCAATTYMTTTRAGTTT) (Quince et al. 2011) in a PCR mixture (25 μL total volume) containing 8.5 μL MQ, 12.5 μL KAPPA HiFi Roche ReadyMix, 0.75 μL forward primer (10 pMol/μL), 0.75 μL reverse primer (10 pMol/μL) and 2.5 μL undiluted DNA template. The thermal cycling scheme was as follows: initial denaturation at 95°C for 3 min, 32 cycles of denaturation at 98°C for 20 s, annealing at 50°C for 30 s, followed by extension at 72°C for 30 s. The final extension was carried out at 72°C for 5 min. PCR products were checked on an Invitrogen 2% agarose E‐Gel to validate blank DNA extractions and PCR negatives. A purification of the PCR product was performed using a 0.9× bead clean‐up using MN beads with a magnetic extractor stamp. Samples were then labelled using the IDT10 library kit in a second PCR mixture (20 μL total volume) containing 5.4 μL MQ, 10 μL KAPPA HiFi Roche ReadyMix, 0.8 μL forward primer (10 pMol/μL), 0.8 μL reverse primer (10 pMol/μL) and 3 μL purified PCR product. The thermal cycling scheme was as follows: initial denaturation at 95°C for 3 min, 8 cycles of denaturation at 98°C for 20 s, annealing at 55°C for 30 s, followed by extension at 72°C for 30 s. The final extension was carried out at 72°C for 5 min. PCR products were then analysed on a Fragment Analyser Agilent 5300 using the DNF‐91033 dsDNA Reagent Kit (35–1500 bp) to confirm fragment length after labelling and calculate equimolar pooling concentrations. Equimolar pooling of each PCR plate into sub pools was carried out on a QIAqility platform. Bead clean‐ups of the sub pools were performed using MN beads and the molarity of the sub pools was subsequently measured on an Agilent Tapestation 4150 to equimolarly combine the sub pools into the final library. Sequencing was performed on a NovaSeq PE250 platform at Baseclear B.V. in Leiden, the Netherlands. In addition to the sponge samples, blank DNA extractions and PCR negatives were also processed and sequenced for contaminant removal.

After removing samples for which no 16S rRNA gene was amplified based on the gel electrophoresis, the obtained demultiplexed reads were further processed using DADA2 on Rstudio Server following the standard tutorial with some adjustments to a NovaSeq workflow (Callahan et al. 2016). The filter and trimming step was performed using a truncation length of 250 and 240 basepairs for the forward and reverse reads respectively, maxN = 0, maxEE = 2 and truncQ = 2. An error model was created using a Loess Error Function model adapted to a NovaSeq workflow. An ASV table was constructed after dereplication of the sequences using the ‘derepFastq’ function and chimeric sequences were removed. During the DADA2 workflow, on average 45% of the reads were removed per sample. Taxonomic assignment was performed using the Silva v138 reference database. The dataset was decontaminated from sequences identified as contaminants from the blank DNA extractions and PCR negatives using the ‘Decontam’ package in R (Davis et al. 2018). Then the data was filtered to remove all eukaryotic, chloroplast and mitochondrial sequences. Due to the deep sequencing and index‐hopping problems caused by the NovaSeq workflow the dataset contained a large number of ASVs which were most likely false positives (Jia et al. 2022). To circumvent this problem, for each sample, we set all read counts for ASVs below a 0.01% threshold to 0. A phylogenetic tree of all ASVs was generated by exporting all reference sequences to qiime2 and building the phylogenetic tree using the ‘phylogeny align‐to‐tree‐mafft‐fasttree’ command to calculate UniFrac distance metrics for prokaryotic beta diversity. The ASV table, taxonomy table, phylogenetic tree of the ASVs and metadata were combined in a Phyloseq object in Rstudio. The raw reads are uploaded to the NCBI Sequence Read Archive under BioProject accession number PRJNA1252920.

### 2.5 Statistical Analyses

All analyses were performed in Rstudio (R version 4.4.1). Rarefaction curves were generated using the ‘rarecurve’ function from the ‘vegan’ package (Oksanen et al. 2024). For beta‐diversity analyses the dataset was transformed to a compositional dataset using the ‘microbiome’ package (Lahti and Shetty 2017). An NMDS was generated using the Bray–Curtis beta diversity metric in ‘phyloseq’ (McMurdie and Holmes 2013) and visualised using ‘ggplot2’ (Wickham 2016). A PERMANOVA was conducted using the ‘adonis2’ function from ‘vegan’ (Oksanen et al. 2024), to assess the marginal effects of sponge species, geography (Marine Ecoregion) and depth. For samples lacking exact depth measurements, the midpoint of the reported depth range was calculated and used as an approximate depth value in the analysis. Further multivariate pairwise post hoc testing between sponge species was performed using the ‘pairwise.adonis’ function from the ‘pairwiseAdonis’ package (Martinez Arbizu 2020). For alpha‐diversity analyses the dataset was rarefied using the ‘rarefy_even_depth’ function from ‘phyloseq’ (McMurdie and Holmes 2013). Alpha diversity measures were estimated with Chao1 (estimated richness), Pielou (evenness) and Shannon (both richness and evenness) indices using the ‘phyloseq’ and ‘vegan’ packages (McMurdie and Holmes 2013; Oksanen et al. 2024). The Shapiro–Wilk test indicated all alpha diversity measures significantly differed from a normal distribution and thus differences in alpha diversity between sponge species were investigated using a Kruskal–Wallis test, followed by a pairwise Wilcoxon test using the ‘agricolae’ package (de Mendiburu 2023). To test for phylogenetic signal in prokaryotic richness across the host phylogeny, Pagels Lambda (Pagel 1999) was calculated using the ‘phytools’ package (Revell 2024). For visualisation purposes a phylogenetic consensus tree was built by generating consensus sequences for each sponge species choosing a 100% threshold to account for all genetic variance within the species (Figure S1), and combined with the average alpha diversity per species (calculated as the mean Shannon diversity index of all samples belonging to each species) and prokaryotic composition (visualised by determining the relative abundance of prokaryotic groups at family level based on the combined read counts per species). To study phylosymbiosis, first a Mantel test was performed using Pearson correlation with 9999 permutations to test for correlations in the distance matrices of the host phylogenetic tree and the prokaryotic community using the ‘vegan’ package (Oksanen et al. 2024). Prokaryotic distance matrices were calculated based on three beta diversity metrics (Bray–Curtis, Weighted UniFrac and Unweighted UniFrac) and the phylogenetic tree distance matrix was based on patristic distances. Secondly, topological congruence of the host phylogenetic tree and prokaryotic dendrogram at ASV level was evaluated using the normalised Robinson Foulds (nRF) distance calculated using the ‘RFmeasures’ function with 999 permutations (Mazel et al. 2018). Prokaryotic dendrograms were constructed using the ‘hclust’ function from the ‘stats’ package, applying the ‘complete’ linkage clustering method to all three beta diversity metrics (Bray–Curtis, Weighted UniFrac and Unweighted UniFrac). Tanglegrams were visualised using the ‘dendextend’ package (Galili 2015) by untangling the phylogenetic tree and prokaryotic dendrogram using a ‘step2side’ approach. A tanglegram approach was also used to test congruence of phylogenetic trees. The full script for data analysis can be found on GitHub (https://github.com/nielsvanderwindt/2025_Petrosiidae‐phylosymbiosis).

## 3 Results

### 3.1 Sample Selection and Sponge Taxonomy

After processing, a total of 169 28S rRNA, 144 18S rRNA and 142 COI sequences belonging to 21 different species of Petrosiidae were obtained. For 124 samples all three barcoding markers were available. Further testing showed considerable congruence between the phylogeny based on 28S rRNA and the concatenated phylogeny (Figure S2). Therefore, all samples for which 28S rRNA gene was obtained, were included in the phylosymbiosis analyses. The phylogenetic tree (Figure S3) indicates that Petrosiidae genera as currently defined based on morphological characteristics do not form monophyletic groups, but are rather polyphyletic. At the species level, most taxa do exhibit well‐defined boundaries (Figure S3), except for the giant barrel sponge clade (represented by *Xestospongia testudinaria* and *Xestospongia muta* in this study; Swierts et al. 2017), and the *Neopetrosia eurystomata* and *Neopetrosia proxima* clade. *Neopetrosia eurystomata* and *N. proxima* cannot be distinguished based on their barcodes, but are separated as species based on morphology and habitat. Similarly, the Caribbean species *Neopetrosia carbonaria* and Indonesian species *Xestospongia viridenigra* are molecularly closely related and morphologically very similar. However, they occur in different oceans and different habitats leading to their classification as distinct species belonging to different genera.

### 3.2 Prokaryotic Assemblages

**FIGURE 1:**
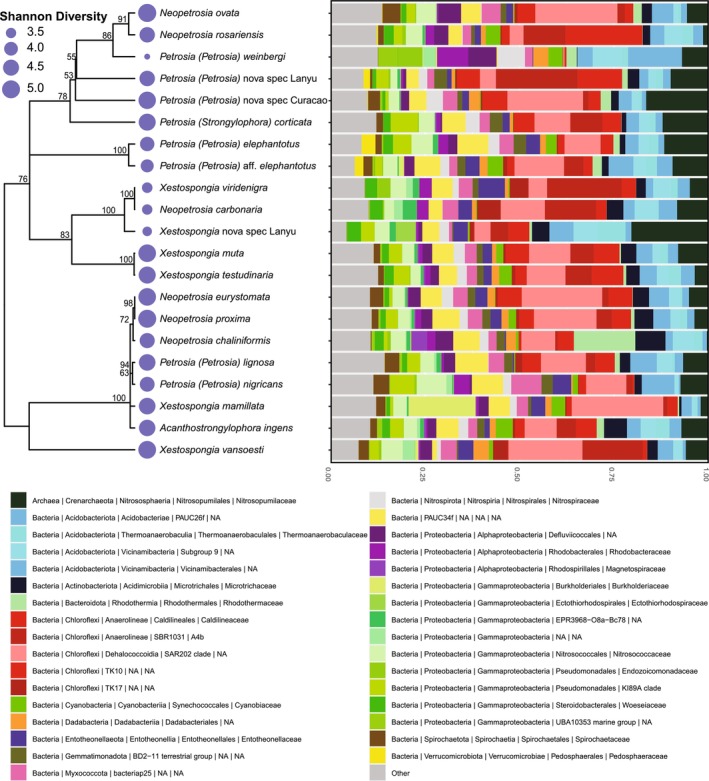
Maximum likelihood phylogenetic tree (28S rRNA) based on the generated consensus sequences of all sequences per species for Petrosiidae included in this study. The blue coloured circles at the tips indicate the average Shannon diversity values of the corresponding species ranging between 3.26 and 5.05. The bar plot represents the community composition at family level of the most abundant prokaryotic taxa of the respective sponge species. Prokaryotic families with a relative abundance below 3% were grouped in ‘Other’.

16S rRNA gene sequencing resulted in a total of 78,827,466 reads remaining after preprocessing and filtering of the dataset. The number of reads per sample ranged from 107,923 to 1,151,495. These reads contained 14,900 unique ASVs present within the sponge samples. Rarefaction curves reached the asymptote for each sample (Figure S4). In total 38 prokaryotic phyla were identified in the sponge samples (Figure 1). The most abundant prokaryotic phyla, based on read counts, were Chloroflexi (29.8%), Proteobacteria (19.5%), Acidobacteriota (14.2%), Crenarchaeota (7.3%) and PAUC34f (5.2%).

**FIGURE 2:**
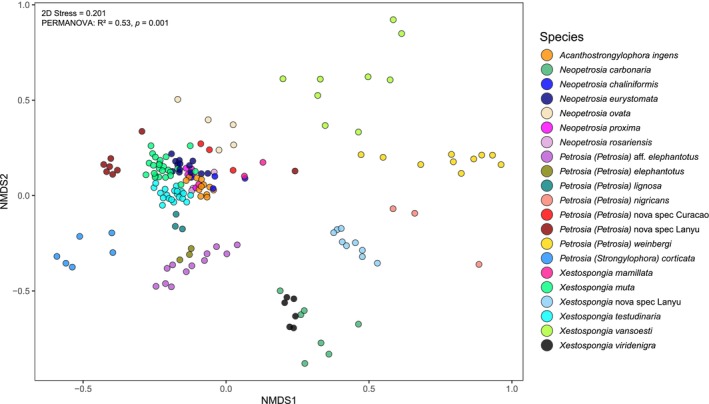
Nonmetric multidimensional scaling (NMDS) based on Bray–Curtis dissimilarities of the prokaryotic communities in Petrosiidae sponges.

Analyses of the beta diversity of the samples show that sponge species are a significant factor in determining prokaryotic community composition within Petrosiidae (PERMANOVA: *R* ^2^ = 0.53, *p* = 0.001; Table S3; Figure 2). Post hoc pairwise comparisons further support this pattern, with only 15 out of 210 comparisons between sponge species being statistically non‐significant (Table S4). Geography as defined by marine ecoregions (PERMANOVA: *R* ^2^ = 0.04, *p* = 0.001; Table S3), and sampling depth (PERMANOVA: *R* ^2^ = 0.01, *p* = 0.001; Table S3) exhibited smaller yet statistically significant effects on community composition.

Similarly, the Kruskal–Wallis test shows significant differences in alpha‐diversity between sponge species based on Shannon, Chao1 and Pielou indices (*p* < 0.001; Table S5). *Petrosia* (*Petrosia*) *weinbergi* exhibited the lowest average Shannon diversity (3.26). In contrast, *Neopetrosia ovata* and *N. proxima* displayed the highest average Shannon diversity values, at 5.05 and 5.01, respectively (Figure S5). The Pielou index followed a very similar pattern as Shannon diversity, underscoring that species with a lower prokaryotic richness typically also show a more uneven distribution of prokaryotic taxa, compared to species with a higher prokaryotic richness (Figure S5). Three out of nine *Xestospongia vansoesti* samples displayed elevated Chao1 (1747–2095) and Shannon (6.06–7.04) index values. These differences could not be attributed to differences in sampling location, environment or phylogeny. The pairwise Wilcoxon rank‐sum test comparing Shannon index between individual sponge species showed 24 out of 210 statistically significant differences (Table S6).

### 3.3 Phylosymbiosis Patterns and Phylogenetic Signal

**FIGURE 3:**
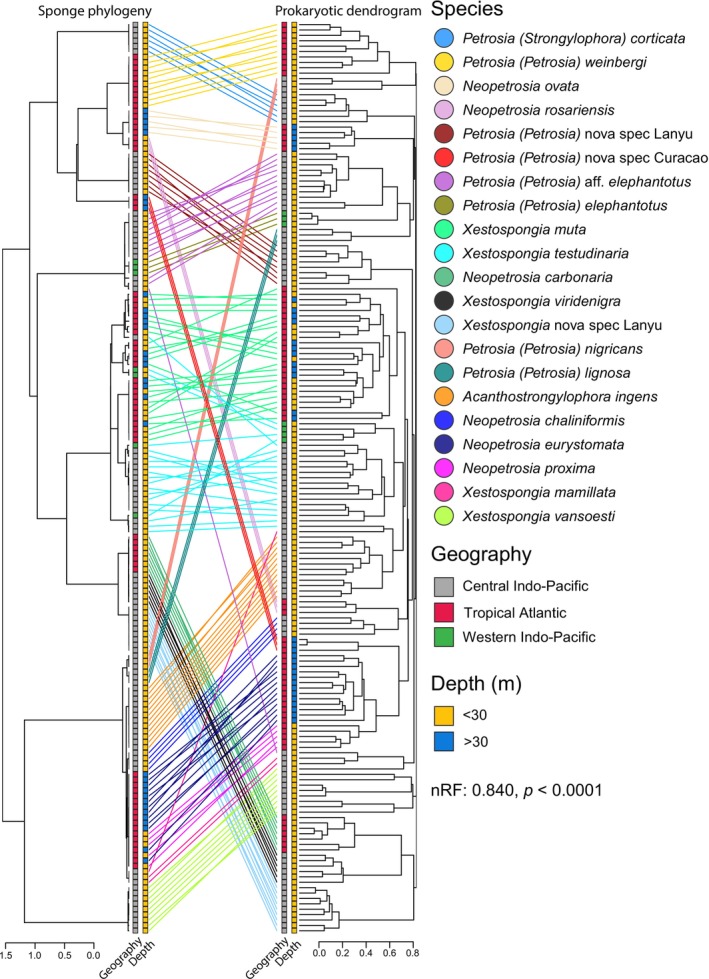
Tanglegram comparing the sponge phylogeny based on 28S rRNA (left) to the prokaryotic dendrogram (right) for Petrosiidae sponges. Congruence between the two trees reflects phylosymbiosis. For each tip, on both sides of the tanglegram, geographic location and depth of the respective sample are represented by a coloured square.

Firstly, distance matrix‐based metrics to study phylosymbiosis patterns (Mantel test) revealed significant positive correlations between phylogenetic distances and prokaryotic beta diversity across all beta‐diversity metrics (Table 1; Figure S6). The presence/absence‐based Unweighted UniFrac metric exhibited a stronger signal (*r*: 0.202, *p* = 0.0001) than the abundance‐based Bray–Curtis and Weighted UniFrac metrics (*r*: 0.191, *p* = 0.0001; *r*: 0.149, *p* = 0.0001). Secondly, the topology‐based metric to study phylosymbiosis patterns (nRF) showed significant congruence between sponge host and prokaryotic dendrogram topologies (Table 1). Here relative abundance‐based metrics showed a stronger signal in topology‐based metrics than the presence/absence metric. The best congruence is found based on Bray–Curtis distance (nRF: 0.840, *p* < 0.0001). The overall pattern in the tanglegram (Figure 3) shows multiple clades of phylogenetically closely related species crossing over to the prokaryotic dendrogram, while maintaining the same arrangement, displaying congruency and showing that phylogenetically similar Petrosiidae species also harbour similar prokaryotic communities. For instance, the clade comprising *Acanthostrongylophora ingens*, *Neopetrosia chaliniformis*, *N. eurystomata*, *N. proxima* , *Xestospongia mamillata* and *X. vansoesti* maintains the same arrangement when crossing from the phylogeny to the prokaryotic dendrogram, showing no entanglement between the species. However, the tanglegram is not fully congruent, and several incongruencies appear to be associated with other factors, such as depth or geography. For instance, *Petrosia* (*Petrosia*) nova spec Curacao exhibits a very different prokaryotic community compared to phylogenetically related species, but clusters with another mesophotic species (*Neopetrosia eurystomata*). Likewise, *Neopetrosia ovata* (occurs deeper than 30 m) and *Neopetrosia rosariensis* (occurs shallow) are closely related species from the Tropical Atlantic; however their prokaryotic communities differ considerably as shown in the dendrogram, highlighting the influence of depth on prokaryotic community structure, as also supported by the PERMANOVA results (*R* ^2^ = 0.01, *p* = 0.001; Table S3). Nevertheless, the explained variance of depth in the PERMANOVA is low and depth does not universally explain the observed patterns in the tanglegram as not all mesophotic species are clustered together. In particular, *N. ovata* remains distinct from the other two mesophotic species (*N. eurystomata* and a new species of *Petrosia* (*Petrosia*)). Additionally, geographic structuring is observed within the giant barrel sponge species complex ( *X. muta* and *X. testudinaria*). While the host phylogeny of samples from multiple geographic locations is intertwined, their prokaryotic communities are separated based on geographical region. This pattern aligns with the significant, but modest effect of geography on community composition (PERMANOVA: *R* ^2^ = 0.04, *p* = 0.001; Table S3). Lastly, we evaluated the effect of phylogenetic tree structure on prokaryotic alpha diversity, assessed using the Shannon index as a measure that incorporates both richness and evenness. Pagel's Lambda shows a relatively strong phylogenetic signal (Lambda: 0.78), suggesting that closely related species tend to exhibit similar levels of prokaryotic richness (Figure 1), however this association was not statistically significant (*p* = 0.10).

**TABLE 1:** Phylosymbiosis metrics for different beta‐diversity metrics.

	Bray–Curtis	Weighted UniFrac	Unweighted UniFrac
Mantel	*r*: 0.191, *p* = 0.0001	*r*: 0.149, *p* = 0.0001	*r*: 0.202, *p* = 0.0001
nRF	0.840, *p* < 0.0001	0.865, *p* < 0.0001	0.877, *p* < 0.0001

## 4 Discussion

In this study we assessed the phylosymbiosis signal within the sponge family Petrosiidae. Our results demonstrate that host identity is the primary driver shaping sponge microbiomes. This is consistent with previous sponge microbiome studies showing that sponge microbiomes exhibit a high degree of host specificity (O'Brien et al. 2019; Webster and Thomas 2016). Further testing revealed evidence of phylosymbiosis within the sponge family Petrosiidae using distance‐based metrics and topological congruence analytical approaches; however the analysis of phylogenetic signal on prokaryotic richness yielded a statistically insignificant result.

Our results are in agreement with previous studies that found phylosymbiosis signal across various sponge taxa. Previous smaller‐scale studies with fewer samples, or those focusing on a broader range of taxonomic diversity typically detect stronger phylosymbiosis signal (Easson and Thacker 2014; O'Brien et al. 2020). In contrast, larger‐scale studies with greater complexity (e.g., Pankey et al. 2022; Thomas et al. 2016) or studies that focus on closely related species or cryptic species often detect weaker phylosymbiosis signal. For instance, phylosymbiosis has been observed at the genus level, such as for *Geodia* species (Schöttner et al. 2013), within a cryptic species complex of *Agelas* (Pankey et al. 2024), and between different genetic clusters of *Petrosia ficiformis* (Díez‐Vives et al. 2020). These findings highlight that phylosymbiosis is detectable across multiple taxonomic levels in sponges. Moreover, previous studies have noted that larger phylogenetic distances increase the chance of observing phylosymbiosis (O'Brien et al. 2020).

In this study, we observe phylosymbiosis through significant congruence in the tanglegram, but we also observe a number of incongruences that appear to be associated with depth or geographical location. The proportion of variance explained by these factors was low, although this is likely due to the heterogeneous nature of the dataset. Many species were endemic, occurred within narrow depth ranges or lacked sufficient biological replication to robustly assess for depth and biogeographical influences. However, previous studies have shown strong effects of both depth (e.g., Busch et al. 2022; Morrow et al. 2016; Paix et al. 2025; Williams et al. 2024) and biogeography (e.g., Cleary et al. 2022; Díez‐Vives et al. 2020; Luter et al. 2015; Swierts et al. 2018) on sponge microbial communities. Therefore, these observations in the tanglegram suggest that while host phylogeny is a key determinant of microbial community structure, local environmental conditions such as depth and geographical location can also contribute. However, our dataset does not show that these factors consistently account for the observed incongruencies. Our results therefore support a primary role of host phylogeny in structuring microbial communities, while acknowledging that additional ecological and host‐related factors can introduce variability that may obscure or even override phylosymbiosis patterns. One hypothesis that follows, consistent with suggestions that greater phylogenetic distances amplify the phylosymbiosis signal (O'Brien et al. 2020), is that environmental and biogeographical factors may play a larger role in shaping microbiomes when host taxa are more closely related. Although our dataset cannot test this directly, it represents an interesting hypothesis for future research. Alternative explanations, including host physiological differences and species‐specific ecological adaptations, should also be considered when interpreting these incongruencies.

While phylosymbiosis is a widespread phenomenon, its underlying mechanisms remain largely unknown, particularly in sponges. Although phylosymbiosis may indicate co‐evolutionary processes or host divergence, it only represents a pattern constrained by specific temporal and spatial contexts (Moran and Sloan 2015; van Opstal and Bordenstein 2019). One likely contributor to phylosymbiosis is the vertical transmission of microbes, which would preserve host‐microbe congruence across evolutionary timescales and has been observed in sponges (Glasl et al. 2024; Paix et al. 2024; Schmitt et al. 2007; Sipkema et al. 2015). However, phylosymbiosis may also result from the horizontal transmission of microbes filtered through phylogenetically congruent traits in a process where there is ecological filtering of microbes (Mazel et al. 2018). Horizontal and vertical transmission are not mutually exclusive, and a mixed mode of transmission may be most common (Björk et al. 2019; de Oliveira et al. 2020; Sipkema et al. 2015). Further research could elaborate on this by investigating which microbial taxa drive phylosymbiosis patterns, which will be instrumental in understanding how and why phylosymbiosis occurs. Additionally, studying these patterns in combination with geographical location and environmental conditions, both of which play crucial roles in structuring microbial communities (Cleary et al. 2022; Easson et al. 2020; Swierts et al. 2018), could improve our understanding of the processes behind phylosymbiosis.

While the normalised Robinson Foulds (nRF) metric is widely used for evaluating topological congruence in phylosymbiosis research (Lim and Bordenstein 2020), it appears to have its limitations in complex datasets. For instance, due to complications which are inherent to sampling, the sponges in this study are collected over large geographic areas, across different time periods and diverse ecological habitats. These factors introduce variability and complexity in the dataset, potentially obscuring phylosymbiosis patterns and skewing nRF values, highlighting the importance of reducing ecological, geographical and temporal variability in the dataset to isolate phylosymbiosis patterns. This is reflected in relatively low, yet statistically significant nRF values (> 0.8) observed in this study, indicating a weak but notable congruence between the sponge phylogeny and the prokaryotic dendrogram. However, visual inspection of the tanglegram shows large sections of congruence, signalling phylosymbiosis despite lower nRF values. It should be noted that visual inspection of a tanglegram is prone to underestimating incongruence (de Vienne 2019). To strengthen our conclusion on the persistence of phylosymbiosis, we corroborated the pattern with additional analyses. A distance‐based Mantel test revealed significant positive correlations between host genetic distance and prokaryotic community dissimilarity. Given the limited number of sponge phylosymbiosis studies however, placing our normalised Robinson‐Foulds (nRF) and Mantel statistics in ecological context remains challenging; future studies should establish a more robust framework for their interpretation. Additionally, phylogenetic signal analysis based on Pagel's Lambda shows strong phylogenetic structuring of prokaryotic alpha diversity, albeit not statistically significant. Similarly, both the nRF and Mantel test were conducted using multiple beta diversity metrics (Bray–Curtis, Unweighted UniFrac and Weighted UniFrac). While results varied slightly depending on the metric, all yielded similar statistically significant outcomes, emphasising the robustness of the observed pattern, even when different metrics may influence phylosymbiosis results (Lim and Bordenstein 2020). Together these methods consistently point towards the same direction, highlighting the importance of a comprehensive approach that combines multiple analytical methods including topological congruence, distance‐based metrics and phylogenetic signal to reinforce the conclusion that phylosymbiosis persists in Petrosiidae sponges.

While the sponge family Petrosiidae serves as a suitable model to study host–microbe interactions because of its diversity and widespread distribution, its taxonomy remains challenging. The current taxonomy has been based on morphological characteristics, but molecular data from both literature and our own studies highlight that the sponge family Petrosiidae is likely polyphyletic rather than monophyletic (Redmond et al. 2007, 2011; van der Sprong et al. 2023, 2025). This reflects a broader issue within the sponge order Haplosclerida where there is a significant discrepancy between molecular and morphological data (Leal et al. 2025; McCormack et al. 2002; Redmond et al. 2011; van der Sprong et al. 2023, 2025). Additionally, the presence of cryptic species, such as the giant barrel sponge complex (Swierts et al. 2017), and species with identical molecular sequences further complicate taxonomic and phylogenetic analyses. These challenges underscore the need for robust and well‐supported phylogenies and taxonomy as a foundation for host–microbiome studies, including phylosymbiosis research. More specifically, while sponge barcoding can be useful for detecting phylosymbiosis patterns, a deeper understanding of the underlying mechanisms and functional implications of sponge phylosymbiosis requires comprehensive insights into sponge host taxonomy and biology.

This study is the first to comprehensively assess phylosymbiosis at the family level at this scale. Our findings demonstrate that phylosymbiosis persists in closely related sponge species, while highlighting the importance of considering environmental and geographical influences on phylosymbiosis patterns. It represents a significant step forward in phylosymbiosis research in sponges, providing valuable insights into the drivers of microbial diversity, particularly within the sponge family Petrosiidae.

## Author Contributions

N.W., B.P., D.S., J.C.B. and N.J.V. conceptualised the research; N.W., B.P., D.S., N.J.V., R.A.‐R., Y.M.H. and K.G.S.N. were involved in resource collection; N.W. and N.J.V. conducted laboratory work; N.W., N.J.V. and B.P. performed formal analyses; N.J.V, D.S. and J.C.B. provided supervision; N.W. wrote the first draft of the manuscript, and all authors edited various versions of the manuscript.

## Funding

This project has received funding from the European Union's Horizon 2020 research and innovation programme under Grant Agreement no. 101000392 (MARBLES). The fieldwork for Curacao, Taiwan, Sri Lanka and Indonesia was financed within NWO‐VIDI with project number 16.161.301 by the Netherlands Organization for Scientific Research (NWO). Fieldwork in Martinique was supported by the European Regional Development Fund (ERDF) and the Territorial Collectivity of Martinique (CTM). Fieldwork in the Red Sea was funded through funding from Kaust (award no. CRG‐1‐814 2012‐BER‐002). The samples of Sri Lanka were collected in collaboration with the National Aquatic Resources Research and Development Agency (NARA) and permission from the Ministry of Fisheries and Aquatic Resources (MFAR). The fieldwork in Indonesia was made possible through BRIN Research Permit number 136/SIP/IV/FR/3/2024. The research in Taiwan was facilitated by the Ministry of Science and Technology (MOST) and the Marine National Parks Headquarters (MNPH), Taiwan to Y.M.H. (MOST 105‐2621‐B‐346‐002 and MNPH104403).

## Disclosure

Benefit‐Sharing Statement: All data produced as part of this project is publicly available. All samples included in this study have been sampled under the required documentation and regulation. A research collaboration was developed with scientists from the countries where samples were collected; all collaborators are included as co‐authors.

## Conflicts of Interest

The authors declare no conflicts of interest.

## Supporting information


**Appendix S1:** mec70186‐sup‐0001‐AppendixS1.docx.

## Data Availability

Raw 16S rRNA reads are uploaded to the NCBI Sequence Read Archive under BioProject accession number PRJNA1252920. Additionally, all molecular barcode sequences were deposited in GenBank under the accession numbers PV350143–PV350311 (28S rRNA), PV351460–PV351603 (18S rRNA) and PV351187–PV351328 (COI). Finally, all code used to analyze the data and prepare the figures has been uploaded to GitHub (https://github.com/nielsvanderwindt/2025_Petrosiidae‐phylosymbiosis), including all information required to run these analyses.
