# Supplementary material for: Host Evolutionary History Drives Prokaryotic Diversity in the Globally Distributed Sponge Family Petrosiidae
Source: Mol Ecol. 2025 Nov 28;34(24):e70186. doi: 10.1111/mec.70186 (PMC12717976; doi:10.1111/mec.70186)
Supplement: Supplementary file 1 — Appendix S1: mec70186‐sup‐0001‐AppendixS1.docx. [file MEC-34-e70186-s001.docx]

Supplementary information for :

**Host evolutionary history drives prokaryotic diversity in the globally distributed sponge family Petrosiidae**

N. van der Windt, B. Paix, K. Biesmeijer, R. Ambo-Rappe, Y.M. Huang, K.G.S. Nirbadha, D. Sipkema, N.J. de Voogd


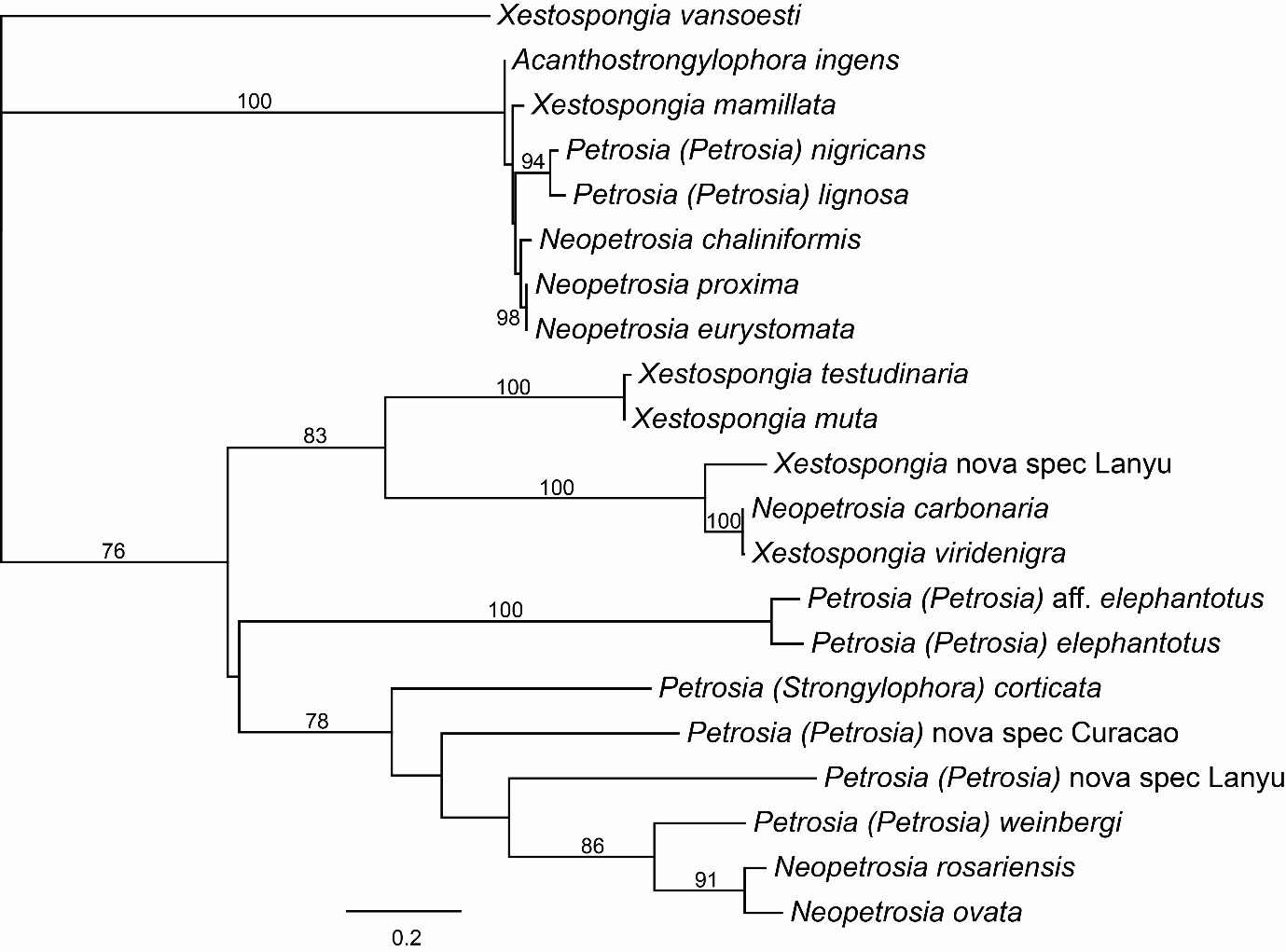


**Figure S1.** Maximum-likelihood phylogeny based on the generated consensus sequences of all sequences per species of 28S rRNA. The phylogeny was constructed using RAxML version 8 (Stamatakis, 2014) in Geneious 2025.0.3 with rapid bootstrapping and search for the best-scoring ML tree (1000 replicates) using the GTR GAMMA I nucleotide model. Numbers on the branches indicate the bootstrap values. Only bootstrap values higher than 75 are displayed.

**
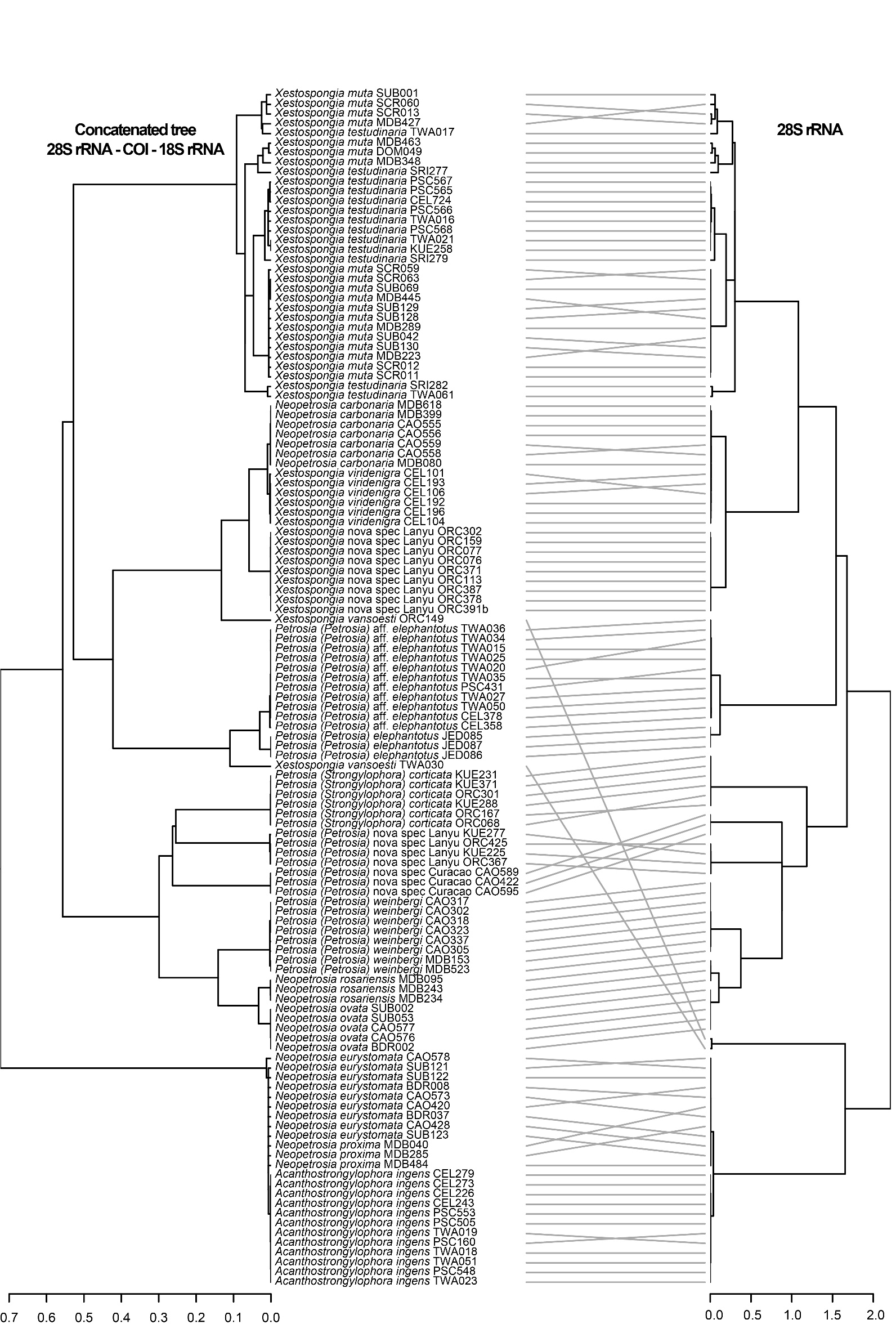
**

**Figure S2** Congruence of the maximum-likelihood phylogenies based on the concatenated markers 28S rRNA, COI and 18S rRNA versus 28S rRNA, only using the samples for which all concatenated markers are available for a fair comparison. Phylogenies were constructed using RAxML version 8 (Stamatakis, 2014) in Geneious 2025.0.3 with rapid bootstrapping and search for the best-scoring ML tree (1000 replicates) using the GTR GAMMA I nucleotide model. Tip labels indicate the species and field ID.


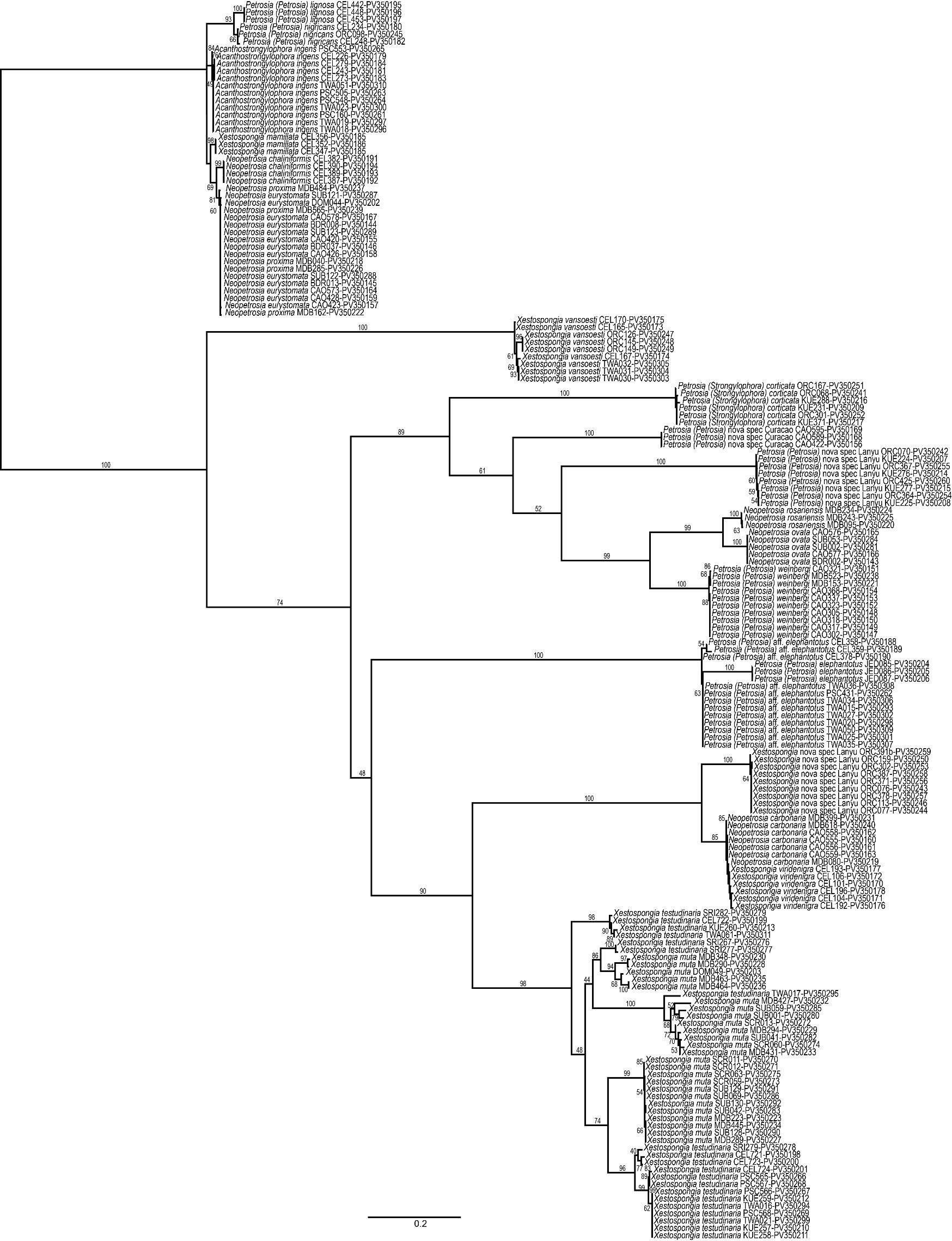


**Figure S3.** Maximum-Likelihood phylogeny of the samples included in this study based on 28S rRNA. The phylogeny was constructed using RAxML version 8 (Stamatakis, 2014) in Geneious 2025.0.3 with rapid bootstrapping and search for the best-scoring ML tree (1000 replicates) using the GTR GAMMA I nucleotide model. Numbers on the branches indicate the bootstrap values. Only bootstrap values higher than 50 or major node support are displayed. Tip labels indicate the sample species followed by the sample ID-GenBank Accession number of the corresponding sequence. A high quality PDF version of this figure is uploaded to <https://github.com/nielsvanderwindt/2025_Petrosiidae-phylosymbiosis>.

**
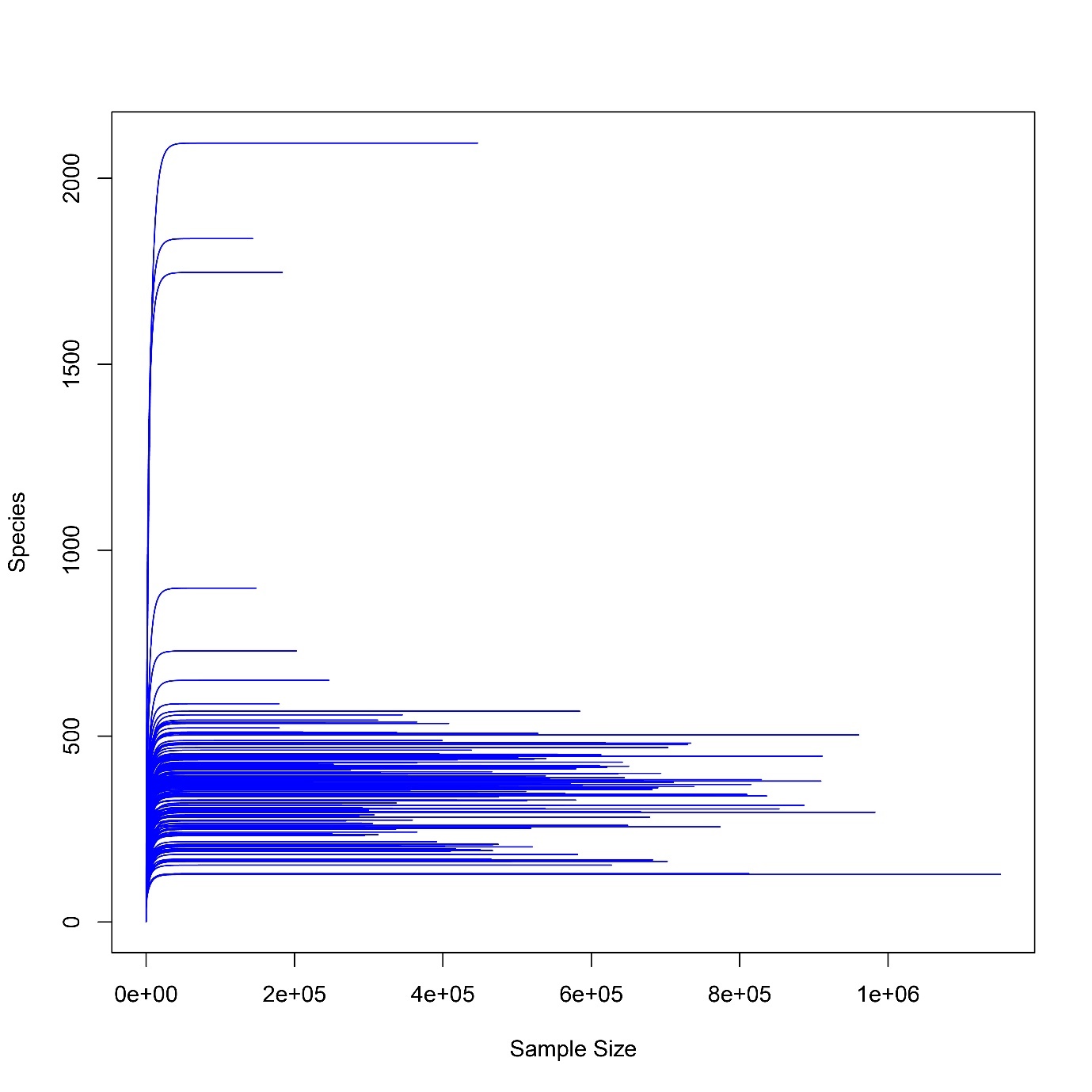
**

**Figure S4.** Rarefaction curves of the 16S rRNA gene reads.

**
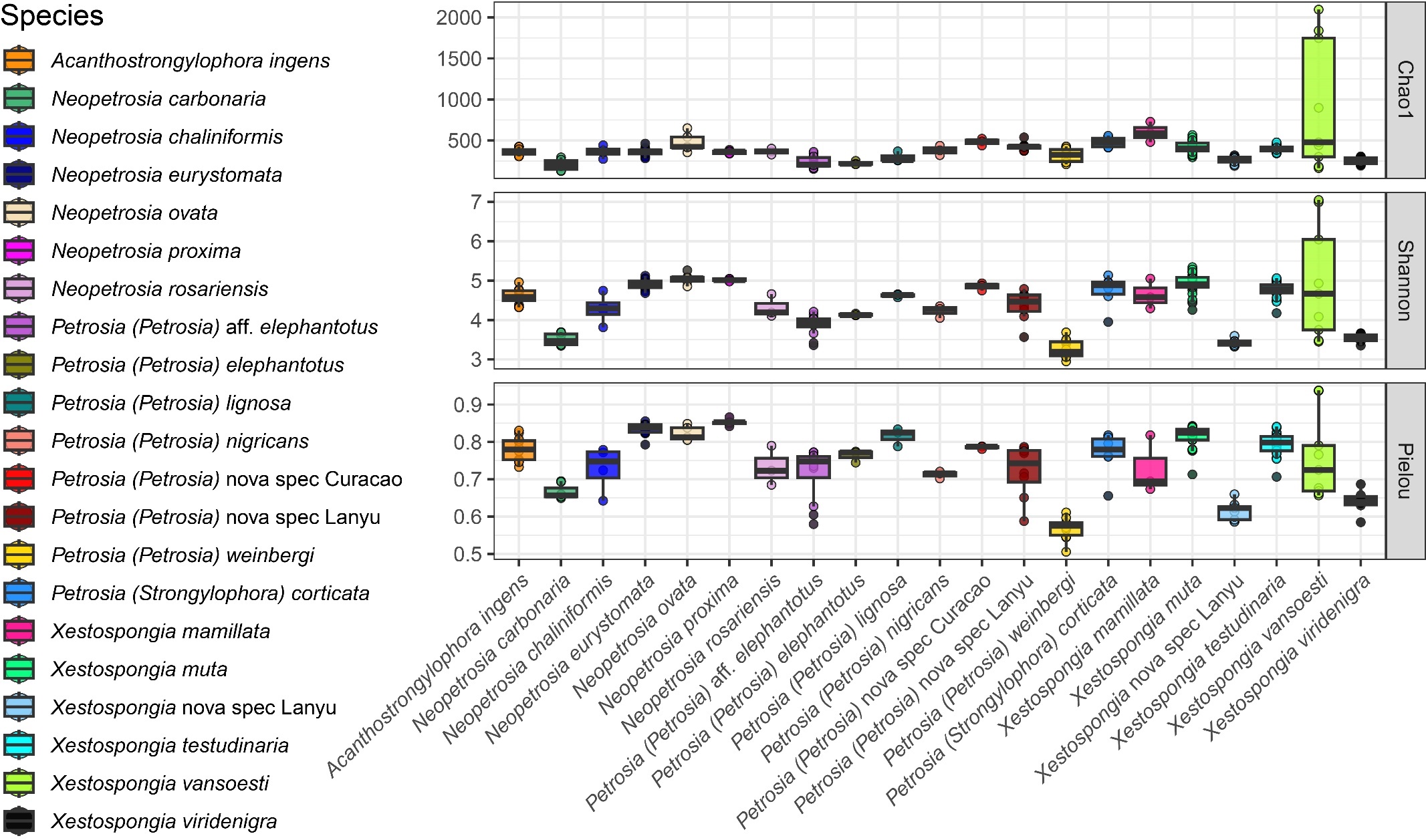
**

**Figure S5.** Alpha diversity analyses of sponge-associated prokaryotic communities.


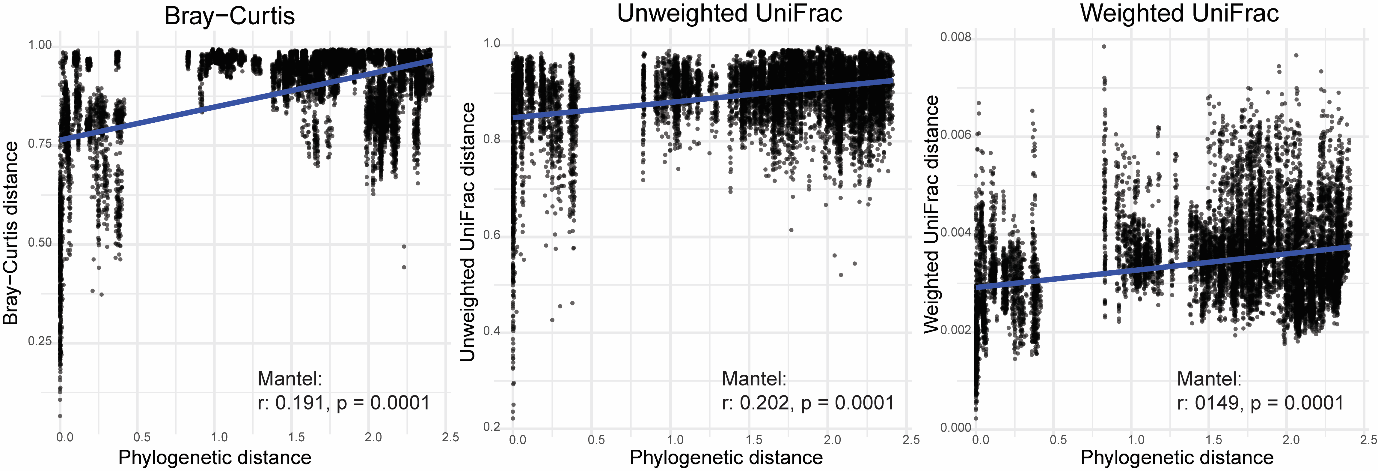


**Figure S6**. Scatterplots of the mantel test correlations between host phylogenetic distance and prokaryotic community dissimilarities of the beta diversity calculated using Bray-Curtis, Unweighted UniFrac and Weighted UniFrac distance metrics.

**Table S1.** Detailed information on the samples included in t53is study. This table can also be found as a spreadsheet on the GitHub page linked to this manuscript (<https://github.com/nielsvanderwindt/2025_Petrosiidae-phylosymbiosis>).

| **extract ID** | **Field nr** | **Museum Voucher** | **Species** | **COI GenBank Accession Number** | **18S GenBank Accession Number** | **28S GenBank Accession Number** | **Country** | **Marine Realm** | **Marine Ecoregion** | **Latitude** | **Longitude** | **Collection Depth Range** | **Approximate Collection Depth** | **Collection Depth (m)** | **Sampling date (YYYY-MM-DD)** |
| --- | --- | --- | --- | --- | --- | --- | --- | --- | --- | --- | --- | --- | --- | --- | --- |
| e1100004418 | KUE259 |  | Xestospongia testudinaria | PV351187 |  | PV350212 | Taiwan | Central Indo-Pacific | South Kuroshio | 22.05638 | 121.5067 | <30m | 15 | 15 | 2019-07-31 |
| e1100004419 | KUE371 |  | Petrosia (Strongylophora) corticata | PV351188 | PV351460 | PV350217 | Taiwan | Central Indo-Pacific | South Kuroshio | 22.08007 | 121.5596 | <30m | 4 | 3-5 | 2019-08-10 |
| e1100004421 | ORC149 |  | Xestospongia vansoesti | PV351189 | PV351461 | PV350249 | Taiwan | Central Indo-Pacific | South Kuroshio | 22.00809 | 121.5947 | <30m | 15 | 15 | 2018-08-12 |
| e1100004422 | ORC364 |  | Petrosia (Petrosia) nova spec Lanyu | PV351190 |  | PV350254 | Taiwan | Central Indo-Pacific | South Kuroshio | 22.00809 | 121.5947 | <30m | 4 | 3-5 | 2018-10-08 |
| e1100004425 | PSC568 |  | Xestospongia testudinaria | PV351191 | PV351462 | PV350269 | Taiwan | Central Indo-Pacific | Southern China | 23.55396 | 119.6423 | <30m | 5 | 5 | 2016-08-07 |
| e1100004426 | JED087 | RMNH.POR.9592 | Petrosia (Petrosia) elephantotus | PV351192 | PV351463 | PV350206 | Saudi Arabia | Western Indo-Pacific | Northern and Central Red Sea | 22.25519 | 39.02564 | <30m | 20 | 20 | 2014-11-10 |
| e1100004430 | KUE260 |  | Xestospongia testudinaria | PV351193 |  | PV350213 | Taiwan | Central Indo-Pacific | South Kuroshio | 22.05638 | 121.5067 | <30m | 15 | 15 | 2019-07-31 |
| e1100004431 | ORC068 |  | Petrosia (Strongylophora) corticata | PV351194 | PV351464 | PV350241 | Taiwan | Central Indo-Pacific | South Kuroshio | 22.00809 | 121.5947 | <30m | 4 | 3-5 | 2018-08-11 |
| e1100004433 | ORC159 |  | Xestospongia nova spec Lanyu | PV351195 | PV351465 | PV350250 | Taiwan | Central Indo-Pacific | South Kuroshio | 22.00809 | 121.5947 | <30m | 4 | 3-5 | 2018-08-13 |
| e1100004434 | ORC367 |  | Petrosia (Petrosia) nova spec Lanyu | PV351196 | PV351466 | PV350255 | Taiwan | Central Indo-Pacific | South Kuroshio | 22.00809 | 121.5947 | <30m | 4 | 3-5 | 2018-10-08 |
| e1100004435 | ORC425 |  | Petrosia (Petrosia) nova spec Lanyu | PV351197 | PV351467 | PV350260 | Taiwan | Central Indo-Pacific | South Kuroshio | 22.00809 | 121.5947 | <30m | 4 | 3-5 | 2018-10-11 |
| e1100004436 | PSC505 |  | Acanthostrongylophora ingens | PV351198 | PV351468 | PV350263 | Taiwan | Central Indo-Pacific | Southern China |  |  | <30m | 6 | 6 | 2016-08-06 |
| e1100004439 | MDB080 |  | Neopetrosia carbonaria | PV351199 | PV351469 | PV350219 | Martinique | Tropical Atlantic | Eastern Caribbean | 14.4934 | -61.0911 | <30m | 13.6 | 13.6 | 2016-09-07 |
| e1100004442 | KUE276 |  | Petrosia (Petrosia) nova spec Lanyu | PV351200 | PV351470 | PV350214 | Taiwan | Central Indo-Pacific | South Kuroshio | 22.07503 | 121.5681 | <30m | 4 | 3-5 | 2019-08-01 |
| e1100004443 | ORC070 |  | Petrosia (Petrosia) nova spec Lanyu | PV351201 |  | PV350242 | Taiwan | Central Indo-Pacific | South Kuroshio | 22.00809 | 121.5947 | <30m | 4 | 3-5 | 2018-08-11 |
| e1100004444 | ORC113 |  | Xestospongia nova spec Lanyu | PV351202 | PV351471 | PV350246 | Taiwan | Central Indo-Pacific | South Kuroshio | 22.00809 | 121.5947 | <30m | 15 | 15 | 2018-08-13 |
| e1100004445 | ORC167 |  | Petrosia (Strongylophora) corticata | PV351203 | PV351472 | PV350251 | Taiwan | Central Indo-Pacific | South Kuroshio | 22.00809 | 121.5947 | <30m | 4 | 3-5 | 2018-08-13 |
| e1100004446 | ORC371 |  | Xestospongia nova spec Lanyu | PV351204 | PV351473 | PV350256 | Taiwan | Central Indo-Pacific | South Kuroshio | 22.00809 | 121.5947 | <30m | 4 | 3-5 | 2018-10-08 |
| e1100004451 | MDB095 |  | Neopetrosia rosariensis | PV351205 | PV351474 | PV350220 | Martinique | Tropical Atlantic | Eastern Caribbean | 14.6386 | -61.1397 | <30m | 12 | 12 | 2016-09-08 |
| e1100004452 | MDB234 |  | Neopetrosia rosariensis | PV351206 | PV351475 | PV350224 | Martinique | Tropical Atlantic | Eastern Caribbean | 14.4421 | -61.0396 | <30m | 15.7 | 15.7 | 2016-09-14 |
| e1100004453 | KUE224 |  | Petrosia (Petrosia) nova spec Lanyu | PV351207 |  | PV350207 | Taiwan | Central Indo-Pacific | South Kuroshio | 22.07503 | 121.5681 | <30m | 4 | 3-5 | 2019-08-01 |
| e1100004454 | KUE277 |  | Petrosia (Petrosia) nova spec Lanyu | PV351208 | PV351476 | PV350215 | Taiwan | Central Indo-Pacific | South Kuroshio | 22.07503 | 121.5681 | <30m | 4 | 3-5 | 2019-08-01 |
| e1100004455 | ORC076 |  | Xestospongia nova spec Lanyu | PV351209 | PV351477 | PV350243 | Taiwan | Central Indo-Pacific | South Kuroshio | 22.00809 | 121.5947 | <30m | 4 | 3-5 | 2018-08-11 |
| e1100004458 | ORC378 |  | Xestospongia nova spec Lanyu | PV351210 | PV351478 | PV350257 | Taiwan | Central Indo-Pacific | South Kuroshio | 22.00809 | 121.5947 | <30m | 4 | 3-5 | 2018-10-09 |
| e1100004460 | PSC548 |  | Acanthostrongylophora ingens | PV351211 | PV351479 | PV350264 | Taiwan | Central Indo-Pacific | Southern China |  |  | <30m | 6 | 6 | 2016-08-06 |
| e1100004465 | KUE225 |  | Petrosia (Petrosia) nova spec Lanyu | PV351212 | PV351480 | PV350208 | Taiwan | Central Indo-Pacific | South Kuroshio | 22.07503 | 121.5681 | <30m | 4 | 3-5 | 2019-08-01 |
| e1100004467 | ORC077 |  | Xestospongia nova spec Lanyu | PV351213 | PV351481 | PV350244 | Taiwan | Central Indo-Pacific | South Kuroshio | 22.00809 | 121.5947 | <30m | 4 | 3-5 | 2018-08-11 |
| e1100004468 | ORC126 |  | Xestospongia vansoesti |  |  | PV350247 | Taiwan | Central Indo-Pacific | South Kuroshio | 22.05138 | 121.5101 | <30m | 15 | 15 | 2018-08-12 |
| e1100004469 | ORC301 |  | Petrosia (Strongylophora) corticata | PV351214 | PV351482 | PV350252 | Taiwan | Central Indo-Pacific | South Kuroshio | 22.00809 | 121.5947 | <30m | 4 | 3-5 | 2018-10-07 |
| e1100004470 | ORC387 |  | Xestospongia nova spec Lanyu | PV351215 | PV351483 | PV350258 | Taiwan | Central Indo-Pacific | South Kuroshio | 22.00809 | 121.5947 | <30m | 4 | 3-5 | 2018-10-09 |
| e1100004472 | PSC553 |  | Acanthostrongylophora ingens | PV351216 | PV351484 | PV350265 | Taiwan | Central Indo-Pacific | Southern China |  |  | <30m | 6 | 6 | 2016-08-06 |
| e1100004475 | MDB153 |  | Petrosia (Petrosia) weinbergi | PV351217 | PV351485 | PV350221 | Martinique | Tropical Atlantic | Eastern Caribbean | 14.4446 | -60.8999 | <30m | 14.7 | 14.7 | 2016-09-10 |
| e1100004476 | MDB243 |  | Neopetrosia rosariensis | PV351218 | PV351486 | PV350225 | Martinique | Tropical Atlantic | Eastern Caribbean | 14.4421 | -61.0396 | <30m | 15.7 | 15.7 | 2016-09-14 |
| e1100004477 | KUE231 |  | Petrosia (Strongylophora) corticata | PV351219 | PV351487 | PV350209 | Taiwan | Central Indo-Pacific | South Kuroshio | 22.00809 | 121.5947 | <30m | 4 | 3-5 | 2019-07-30 |
| e1100004481 | ORC302 |  | Xestospongia nova spec Lanyu | PV351220 | PV351488 | PV350253 | Taiwan | Central Indo-Pacific | South Kuroshio | 22.00809 | 121.5947 | <30m | 4 | 3-5 | 2018-10-07 |
| e1100004484 | PSC565 |  | Xestospongia testudinaria | PV351221 | PV351489 | PV350266 | Taiwan | Central Indo-Pacific | Southern China | 23.55396 | 119.6423 | <30m | 5 | 5 | 2016-08-07 |
| e1100004487 | MDB162 |  | Neopetrosia proxima |  | PV351490 | PV350222 | Martinique | Tropical Atlantic | Eastern Caribbean | 14.4446 | -60.8999 | <30m | 18.6 | 18.6 | 2016-09-10 |
| e1100004489 | KUE257 |  | Xestospongia testudinaria | PV351222 |  | PV350210 | Taiwan | Central Indo-Pacific | South Kuroshio | 22.05638 | 121.5067 | <30m | 15 | 15 | 2019-07-31 |
| e1100004492 | ORC145 |  | Xestospongia vansoesti |  | PV351491 | PV350248 | Taiwan | Central Indo-Pacific | South Kuroshio | 22.05138 | 121.5101 | <30m | 15 | 15 | 2018-08-12 |
| e1100004494 | ORC391b |  | Xestospongia nova spec Lanyu | PV351223 | PV351492 | PV350259 | Taiwan | Central Indo-Pacific | South Kuroshio | 22.00809 | 121.5947 | <30m | 4 | 3-5 | 2018-10-09 |
| e1100004495 | PSC160 |  | Acanthostrongylophora ingens | PV351224 | PV351493 | PV350261 | Taiwan | Central Indo-Pacific | Southern China | 23.25597 | 119.5102 | <30m | 4 | 4 | 2016-07-22 |
| e1100004496 | PSC566 |  | Xestospongia testudinaria | PV351225 | PV351494 | PV350267 | Taiwan | Central Indo-Pacific | Southern China | 23.55396 | 119.6423 | <30m | 5 | 5 | 2016-08-07 |
| e1100004497 | JED085 | RMNH.POR.9590 | Petrosia (Petrosia) elephantotus | PV351226 | PV351495 | PV350204 | Saudi Arabia | Western Indo-Pacific | Northern and Central Red Sea | 22.25519 | 39.02564 | <30m | 20 | 20 | 2014-11-10 |
| e1100004501 | KUE258 |  | Xestospongia testudinaria | PV351227 | PV351496 | PV350211 | Taiwan | Central Indo-Pacific | South Kuroshio | 22.05638 | 121.5067 | <30m | 15 | 15 | 2019-07-31 |
| e1100004502 | KUE288 |  | Petrosia (Strongylophora) corticata | PV351228 | PV351497 | PV350216 | Taiwan | Central Indo-Pacific | South Kuroshio | 22.08171 | 121.5113 | <30m | 4 | 3-5 | 2019-08-02 |
| e1100004503 | ORC098 |  | Petrosia (Petrosia) nigricans |  | PV351498 | PV350245 | Taiwan | Central Indo-Pacific | South Kuroshio | 22.00809 | 121.5947 | <30m | 4 | 3-5 | 2018-08-12 |
| e1100004507 | PSC431 |  | Petrosia (Petrosia) aff. elephantotus | PV351229 | PV351499 | PV350262 | Taiwan | Central Indo-Pacific | Southern China | 23.59397 | 119.5082 | <30m | 12 | 12 | 2016-08-04 |
| e1100004508 | PSC567 |  | Xestospongia testudinaria | PV351230 | PV351500 | PV350268 | Taiwan | Central Indo-Pacific | Southern China | 23.55396 | 119.6423 | <30m | 5 | 5 | 2016-08-07 |
| e1100004509 | JED086 | RMNH.POR.9591 | Petrosia (Petrosia) elephantotus | PV351231 | PV351501 | PV350205 | Saudi Arabia | Western Indo-Pacific | Northern and Central Red Sea | 22.25519 | 39.02564 | <30m | 20 | 20 | 2014-11-10 |
| e1100004510 | MDB040 |  | Neopetrosia proxima | PV351232 | PV351502 | PV350218 | Martinique | Tropical Atlantic | Eastern Caribbean | 14.5333 | -61.0879 | <30m | 13 | 13 | 2016-09-06 |
| e1100004511 | MDB223 |  | Xestospongia muta | PV351233 | PV351503 | PV350223 | Martinique | Tropical Atlantic | Eastern Caribbean | 14.4421 | -61.0396 | <30m | 16 | 16 | 2016-09-14 |
| e1100004514 | MDB445 |  | Xestospongia muta | PV351234 | PV351504 | PV350234 | Martinique | Tropical Atlantic | Eastern Caribbean | 14.9144 | -61.149 | >30m | 75 | 60-90 | 2016-09-25 |
| e1100004518 | CAO368 |  | Petrosia (Petrosia) weinbergi | PV351235 |  | PV350154 | Curacao | Tropical Atlantic | Southern Caribbean | 12.06531 | -68.8602 | <30m | 15 |  | 2022-04-24 |
| e1100004519 | CAO422 |  | Petrosia (Petrosia) nova spec Curacao | PV351236 | PV351505 | PV350156 | Curacao | Tropical Atlantic | Southern Caribbean | 12.08319 | -68.8973 | >30m | 138 | 138 | 2022-04-23 |
| e1100004520 | CAO556 |  | Neopetrosia carbonaria | PV351237 | PV351506 | PV350161 | Curacao | Tropical Atlantic | Southern Caribbean | 12.07071 | -68.86 | <30m | 1 | 1 | 2022-04-29 |
| e1100004524 | CEL378 |  | Petrosia (Petrosia) aff. elephantotus | PV351238 | PV351507 | PV350190 | Indonesia | Central Indo-Pacific | Sulawesi Sea/Makassar Strait | -4.96928 | 119.3282 | <30m | 10 | 10 | 2018-05-07 |
| e1100004525 | MDB285 |  | Neopetrosia proxima | PV351239 | PV351508 | PV350226 | Martinique | Tropical Atlantic | Eastern Caribbean | 14.4407 | -61.0291 | <30m | 15 | 15-18 | 2016-09-15 |
| e1100004526 | MDB463 |  | Xestospongia muta | PV351240 | PV351509 | PV350235 | Martinique | Tropical Atlantic | Eastern Caribbean | 14.9144 | -61.149 | >30m | 75 | 60-90 | 2016-09-26 |
| e1100004527 | MDB565 |  | Neopetrosia proxima |  | PV351510 | PV350239 | Martinique | Tropical Atlantic | Eastern Caribbean | 14.5538 | -61.0261 | <30m | 15 |  | 2016-10-06 |
| e1100004529 | CAO323 |  | Petrosia (Petrosia) weinbergi | PV351241 | PV351511 | PV350152 | Curacao | Tropical Atlantic | Southern Caribbean | 12.06531 | -68.8602 | <30m | 17.4 | 17.4 | 2022-04-24 |
| e1100004531 | CAO423 |  | Neopetrosia eurystomata |  | PV351512 | PV350157 | Curacao | Tropical Atlantic | Southern Caribbean | 12.08319 | -68.8973 | >30m | 115 | 115 | 2022-04-23 |
| e1100004532 | CAO558 |  | Neopetrosia carbonaria | PV351242 | PV351513 | PV350162 | Curacao | Tropical Atlantic | Southern Caribbean | 12.07071 | -68.86 | <30m | 1 | 1 | 2022-04-29 |
| e1100004534 | CEL243 |  | Acanthostrongylophora ingens | PV351243 | PV351514 | PV350181 | Indonesia | Central Indo-Pacific | Sulawesi Sea/Makassar Strait | -5.12543 | 119.3437 | <30m | 13 | 13 | 2018-05-02 |
| e1100004536 | CEL382 |  | Neopetrosia chaliniformis |  | PV351515 | PV350191 | Indonesia | Central Indo-Pacific | Sulawesi Sea/Makassar Strait | -4.96928 | 119.3282 | <30m | 2 | 2 | 2018-05-07 |
| e1100004537 | MDB289 |  | Xestospongia muta | PV351244 | PV351516 | PV350227 | Martinique | Tropical Atlantic | Eastern Caribbean | 14.4407 | -61.0291 | <30m | 15 | 15-18 | 2016-09-15 |
| e1100004538 | MDB464 |  | Xestospongia muta | PV351245 |  | PV350236 | Martinique | Tropical Atlantic | Eastern Caribbean | 14.9144 | -61.149 | >30m | 75 | 60-90 | 2016-09-26 |
| e1100004539 | MDB618 |  | Neopetrosia carbonaria | PV351246 | PV351517 | PV350240 | Martinique | Tropical Atlantic | Eastern Caribbean | 14.5763 | -61.0548 | <30m | 7.1 | 7.1 | 2016-10-09 |
| e1100004540 | CAO317 |  | Petrosia (Petrosia) weinbergi | PV351247 | PV351518 | PV350149 | Curacao | Tropical Atlantic | Southern Caribbean | 12.06531 | -68.8602 | <30m | 15 |  | 2022-04-24 |
| e1100004544 | CAO559 |  | Neopetrosia carbonaria | PV351248 | PV351519 | PV350163 | Curacao | Tropical Atlantic | Southern Caribbean | 12.07071 | -68.86 | <30m | 1 | 1 | 2022-04-29 |
| e1100004547 | CEL347 |  | Xestospongia mamillata |  |  | PV350185 | Indonesia | Central Indo-Pacific | Sulawesi Sea/Makassar Strait | -4.92538 | 119.3982 | <30m | 10 | 10 | 2018-05-06 |
| e1100004548 | CEL387 |  | Neopetrosia chaliniformis |  | PV351520 | PV350192 | Indonesia | Central Indo-Pacific | Sulawesi Sea/Makassar Strait | -4.96928 | 119.3282 | <30m | 2 | 2 | 2018-05-07 |
| e1100004549 | MDB290 |  | Xestospongia muta | PV351249 |  | PV350228 | Martinique | Tropical Atlantic | Eastern Caribbean | 14.4407 | -61.0291 | <30m | 15 | 15-18 | 2016-09-15 |
| e1100004550 | MDB484 |  | Neopetrosia proxima | PV351250 | PV351521 | PV350237 | Martinique | Tropical Atlantic | Eastern Caribbean | 14.5185 | -61.0977 | <30m | 11.7 | 11.7 | 2016-09-30 |
| e1100004552 | CAO318 |  | Petrosia (Petrosia) weinbergi | PV351251 | PV351522 | PV350150 | Curacao | Tropical Atlantic | Southern Caribbean | 12.06531 | -68.8602 | <30m | 15 |  | 2022-04-24 |
| e1100004557 | CEL192 |  | Xestospongia viridenigra | PV351252 | PV351523 | PV350176 | Indonesia | Central Indo-Pacific | Sulawesi Sea/Makassar Strait | -5.04839 | 119.3292 | <30m | 1 | 1 | 2018-04-30 |
| e1100004558 | CEL248 |  | Petrosia (Petrosia) nigricans |  | PV351524 | PV350182 | Indonesia | Central Indo-Pacific | Sulawesi Sea/Makassar Strait | -5.12543 | 119.3437 | <30m | 13 | 13 | 2018-05-02 |
| e1100004559 | CEL352 |  | Xestospongia mamillata |  |  | PV350186 | Indonesia | Central Indo-Pacific | Sulawesi Sea/Makassar Strait | -4.92538 | 119.3982 | <30m | 10 | 10 | 2018-05-06 |
| e1100004560 | CEL389 |  | Neopetrosia chaliniformis |  | PV351525 | PV350193 | Indonesia | Central Indo-Pacific | Sulawesi Sea/Makassar Strait | -4.96928 | 119.3282 | <30m | 2 | 2 | 2018-05-07 |
| e1100004561 | MDB294 |  | Xestospongia muta | PV351253 |  | PV350229 | Martinique | Tropical Atlantic | Eastern Caribbean | 14.4407 | -61.0291 | <30m | 15 | 15-18 | 2016-09-15 |
| e1100004563 | CAO302 |  | Petrosia (Petrosia) weinbergi | PV351254 | PV351526 | PV350147 | Curacao | Tropical Atlantic | Southern Caribbean | 12.06865 | -68.8623 | <30m | 25.5 | 25.5 | 2022-04-20 |
| e1100004567 | CAO426 |  | Neopetrosia eurystomata |  | PV351527 | PV350158 | Curacao | Tropical Atlantic | Southern Caribbean | 12.08319 | -68.8973 | >30m | 113 | 113 | 2022-04-23 |
| e1100004568 | CEL101 |  | Xestospongia viridenigra | PV351255 | PV351528 | PV350170 | Indonesia | Central Indo-Pacific | Sulawesi Sea/Makassar Strait | -4.85673 | 119.3847 | <30m | 1 | 1 | 2018-04-27 |
| e1100004569 | CEL193 |  | Xestospongia viridenigra | PV351256 | PV351529 | PV350177 | Indonesia | Central Indo-Pacific | Sulawesi Sea/Makassar Strait | -5.04839 | 119.3292 | <30m | 1 | 1 | 2018-04-30 |
| e1100004571 | CEL356 |  | Xestospongia mamillata |  |  | PV350187 | Indonesia | Central Indo-Pacific | Sulawesi Sea/Makassar Strait | -4.92538 | 119.3982 | <30m | 10 | 10 | 2018-05-06 |
| e1100004572 | CEL390 |  | Neopetrosia chaliniformis |  | PV351530 | PV350194 | Indonesia | Central Indo-Pacific | Sulawesi Sea/Makassar Strait | -4.96928 | 119.3282 | <30m | 2 | 2 | 2018-05-07 |
| e1100004573 | MDB348 |  | Xestospongia muta | PV351257 | PV351531 | PV350230 | Martinique | Tropical Atlantic | Eastern Caribbean | 14.6462 | -60.8508 | <30m | 17 | 16.9-30 | 2016-09-18 |
| e1100004574 | MDB523 |  | Petrosia (Petrosia) weinbergi | PV351258 | PV351532 | PV350238 | Martinique | Tropical Atlantic | Eastern Caribbean | 14.7867 | -61.2184 | <30m | 15 |  | 2016-10-03 |
| e1100004578 | CAO420 |  | Neopetrosia eurystomata | PV351259 | PV351533 | PV350155 | Curacao | Tropical Atlantic | Southern Caribbean | 12.08319 | -68.8973 | >30m | 101 | 101 | 2022-04-23 |
| e1100004579 | CAO428 |  | Neopetrosia eurystomata | PV351260 | PV351534 | PV350159 | Curacao | Tropical Atlantic | Southern Caribbean | 12.08319 | -68.8973 | >30m | 114 | 114 | 2022-04-23 |
| e1100004580 | CEL104 |  | Xestospongia viridenigra | PV351261 | PV351535 | PV350171 | Indonesia | Central Indo-Pacific | Sulawesi Sea/Makassar Strait | -4.85673 | 119.3847 | <30m | 1 | 1 | 2018-04-27 |
| e1100004581 | CEL196 |  | Xestospongia viridenigra | PV351262 | PV351536 | PV350178 | Indonesia | Central Indo-Pacific | Sulawesi Sea/Makassar Strait | -5.04839 | 119.3292 | <30m | 1 | 1 | 2018-04-30 |
| e1100004582 | CEL273 |  | Acanthostrongylophora ingens | PV351263 | PV351537 | PV350183 | Indonesia | Central Indo-Pacific | Sulawesi Sea/Makassar Strait | -5.12543 | 119.3437 | <30m | 14.3 | 14.3 | 2018-05-03 |
| e1100004583 | CEL358 |  | Petrosia (Petrosia) aff. elephantotus | PV351264 | PV351538 | PV350188 | Indonesia | Central Indo-Pacific | Sulawesi Sea/Makassar Strait | -4.92538 | 119.3982 | <30m | 10 | 10 | 2018-05-06 |
| e1100004585 | MDB427 |  | Xestospongia muta | PV351265 | PV351539 | PV350232 | Martinique | Tropical Atlantic | Eastern Caribbean | 14.9178 | -61.1487 | >30m | 75 | 60-90 | 2016-09-25 |
| e1100004588 | CAO321 |  | Petrosia (Petrosia) weinbergi | PV351266 |  | PV350151 | Curacao | Tropical Atlantic | Southern Caribbean | 12.06865 | -68.8623 | <30m | 14 | 14 | 2022-04-20 |
| e1100004592 | CEL106 |  | Xestospongia viridenigra | PV351267 | PV351540 | PV350172 | Indonesia | Central Indo-Pacific | Sulawesi Sea/Makassar Strait | -4.85673 | 119.3847 | <30m | 1 | 1 | 2018-04-27 |
| e1100004593 | CEL226 |  | Acanthostrongylophora ingens | PV351268 | PV351541 | PV350179 | Indonesia | Central Indo-Pacific | Sulawesi Sea/Makassar Strait | -5.12543 | 119.3437 | <30m | 16 | 16 | 2018-05-02 |
| e1100004594 | CEL279 |  | Acanthostrongylophora ingens | PV351269 | PV351542 | PV350184 | Indonesia | Central Indo-Pacific | Sulawesi Sea/Makassar Strait | -5.12543 | 119.3437 | <30m | 10.7 | 10.7 | 2018-05-03 |
| e1100004595 | CEL359 |  | Petrosia (Petrosia) aff. elephantotus |  | PV351543 | PV350189 | Indonesia | Central Indo-Pacific | Sulawesi Sea/Makassar Strait | -4.92538 | 119.3982 | <30m | 16 | 16 | 2018-05-06 |
| e1100004597 | MDB431 |  | Xestospongia muta | PV351270 |  | PV350233 | Martinique | Tropical Atlantic | Eastern Caribbean | 14.9178 | -61.1487 | >30m | 75 | 60-90 | 2016-09-25 |
| e1100004599 | CAO305 |  | Petrosia (Petrosia) weinbergi | PV351271 | PV351544 | PV350148 | Curacao | Tropical Atlantic | Southern Caribbean | 12.06865 | -68.8623 | <30m | 15.8 | 15.8 | 2022-04-20 |
| e1100004601 | CAO337 |  | Petrosia (Petrosia) weinbergi | PV351272 | PV351545 | PV350153 | Curacao | Tropical Atlantic | Southern Caribbean | 12.37496 | -69.1587 | <30m | 15.6 | 15.6 | 2022-04-26 |
| e1100004603 | CAO555 |  | Neopetrosia carbonaria | PV351273 | PV351546 | PV350160 | Curacao | Tropical Atlantic | Southern Caribbean | 12.07071 | -68.86 | <30m | 1 | 1 | 2022-04-29 |
| e1100004605 | CEL234 |  | Petrosia (Petrosia) nigricans |  | PV351547 | PV350180 | Indonesia | Central Indo-Pacific | Sulawesi Sea/Makassar Strait | -5.12543 | 119.3437 | <30m | 13.6 | 13.6 | 2018-05-02 |
| e1100004611 | SRI267 |  | Xestospongia testudinaria | PV351274 |  | PV350276 | Sri Lanka | Western Indo-Pacific | South India and Sri Lanka | 8.29155 | 79.74012 | <30m | 16 | 16 | 2019-04-23 |
| e1100004612 | SUB121 |  | Neopetrosia eurystomata | PV351275 | PV351548 | PV350287 | Curacao | Tropical Atlantic | Southern Caribbean | 12.08319 | -68.8973 | >30m | 85 | 85 | 2018-11 |
| e1100004614 | SCR011 |  | Xestospongia muta | PV351276 | PV351549 | PV350270 | Curacao | Tropical Atlantic | Southern Caribbean | 12.08456 | -68.8983 | >30m | 52 | 52 | 2015-11 |
| e1100004618 | TWA018 |  | Acanthostrongylophora ingens | PV351277 | PV351550 | PV350296 | Taiwan | Central Indo-Pacific | Southern China | 23.53745 | 119.5456 | <30m | 5 | 5 | 2023-04-18 |
| e1100004619 | TWA031 |  | Xestospongia vansoesti |  | PV351551 | PV350304 | Taiwan | Central Indo-Pacific | Southern China | 23.25597 | 119.5102 | <30m | 10 | 10 | 2023-04-21 |
| e1100004622 | CEL453 |  | Petrosia (Petrosia) lignosa |  | PV351552 | PV350197 | Indonesia | Central Indo-Pacific | Sulawesi Sea/Makassar Strait | -4.70905 | 118.9646 | <30m | 20 |  | 2018-05-09 |
| e1100004623 | SRI277 |  | Xestospongia testudinaria | PV351278 | PV351553 | PV350277 | Sri Lanka | Western Indo-Pacific | South India and Sri Lanka | 8.29155 | 79.74012 | <30m | 13 | 13 | 2019-04-26 |
| e1100004624 | SUB122 |  | Neopetrosia eurystomata | PV351279 | PV351554 | PV350288 | Curacao | Tropical Atlantic | Southern Caribbean | 12.08319 | -68.8973 | >30m | 102 | 102 | 2018-11 |
| e1100004626 | SCR012 |  | Xestospongia muta | PV351280 | PV351555 | PV350271 | Curacao | Tropical Atlantic | Southern Caribbean | 12.08456 | -68.8983 | >30m | 48 | 48 | 2015-11 |
| e1100004630 | TWA019 |  | Acanthostrongylophora ingens | PV351281 | PV351556 | PV350297 | Taiwan | Central Indo-Pacific | Southern China | 23.53745 | 119.5456 | <30m | 5 | 5 | 2023-04-18 |
| e1100004631 | TWA032 |  | Xestospongia vansoesti |  | PV351557 | PV350305 | Taiwan | Central Indo-Pacific | Southern China | 23.25597 | 119.5102 | <30m | 10 | 10 | 2023-04-21 |
| e1100004634 | CEL721 |  | Xestospongia testudinaria | PV351282 |  | PV350198 | Indonesia | Central Indo-Pacific | Sulawesi Sea/Makassar Strait | -5.12543 | 119.3437 | <30m | 15 | 15 | 2018-04-28 |
| e1100004635 | SRI279 |  | Xestospongia testudinaria | PV351283 | PV351558 | PV350278 | Sri Lanka | Western Indo-Pacific | South India and Sri Lanka | 8.29155 | 79.74012 | <30m | 13 | 13 | 2019-04-26 |
| e1100004636 | SUB123 |  | Neopetrosia eurystomata | PV351284 | PV351559 | PV350289 | Curacao | Tropical Atlantic | Southern Caribbean | 12.08319 | -68.8973 | >30m | 91 | 91 | 2018-11 |
| e1100004638 | SCR013 |  | Xestospongia muta | PV351285 | PV351560 | PV350272 | Curacao | Tropical Atlantic | Southern Caribbean | 12.08456 | -68.8983 | >30m | 48 | 48 | 2015-11 |
| e1100004642 | TWA020 |  | Petrosia (Petrosia) aff. elephantotus | PV351286 | PV351561 | PV350298 | Taiwan | Central Indo-Pacific | Southern China | 23.53745 | 119.5456 | <30m | 5 | 5 | 2023-04-18 |
| e1100004643 | TWA034 |  | Petrosia (Petrosia) aff. elephantotus | PV351287 | PV351562 | PV350306 | Taiwan | Central Indo-Pacific | Southern China | 23.25597 | 119.5102 | <30m | 13 | 13 | 2023-04-21 |
| e1100004646 | CEL722 |  | Xestospongia testudinaria | PV351288 |  | PV350199 | Indonesia | Central Indo-Pacific | Sulawesi Sea/Makassar Strait | -5.12543 | 119.3437 | <30m | 14.6 | 14.6 | 2018-04-28 |
| e1100004647 | SRI282 |  | Xestospongia testudinaria | PV351289 | PV351563 | PV350279 | Sri Lanka | Western Indo-Pacific | South India and Sri Lanka | 8.29155 | 79.74012 | <30m | 12 | 12 | 2019-04-26 |
| e1100004648 | SUB128 |  | Xestospongia muta | PV351290 | PV351564 | PV350290 | Curacao | Tropical Atlantic | Southern Caribbean | 12.06531 | -68.8602 | <30m | 26 | 26 | 2018-11 |
| e1100004650 | SCR059 |  | Xestospongia muta | PV351291 | PV351565 | PV350273 | Curacao | Tropical Atlantic | Southern Caribbean | 12.08456 | -68.8983 | <30m | 27 | 27 | 2015-11 |
| e1100004654 | TWA021 |  | Xestospongia testudinaria | PV351292 | PV351566 | PV350299 | Taiwan | Central Indo-Pacific | Southern China | 23.53745 | 119.5456 | <30m | 5 | 5 | 2023-04-18 |
| e1100004655 | TWA035 |  | Petrosia (Petrosia) aff. elephantotus | PV351293 | PV351567 | PV350307 | Taiwan | Central Indo-Pacific | Southern China | 23.25597 | 119.5102 | <30m | 13 | 13 | 2023-04-21 |
| e1100004658 | CEL723 |  | Xestospongia testudinaria | PV351294 |  | PV350200 | Indonesia | Central Indo-Pacific | Sulawesi Sea/Makassar Strait | -5.12543 | 119.3437 | <30m | 15.1 | 15.1 | 2018-04-28 |
| e1100004659 | SUB041 |  | Xestospongia muta | PV351295 |  | PV350282 | Curacao | Tropical Atlantic | Southern Caribbean | 11.98713 | -68.6474 | >30m | 104 | 104 | 2018-08-29 |
| e1100004660 | SUB129 |  | Xestospongia muta | PV351296 | PV351568 | PV350291 | Curacao | Tropical Atlantic | Southern Caribbean | 12.06531 | -68.8602 | <30m | 28 | 28 | 2018-11 |
| e1100004661 | BDR002 |  | Neopetrosia ovata | PV351297 | PV351569 | PV350143 | Bonaire | Tropical Atlantic | Southern Caribbean | 11.9821 | -68.6452 | >30m | 149 | 149 | 2013-05-27 |
| e1100004662 | SCR060 |  | Xestospongia muta | PV351298 | PV351570 | PV350274 | Curacao | Tropical Atlantic | Southern Caribbean | 12.08456 | -68.8983 | <30m | 27 | 27 | 2015-11 |
| e1100004666 | TWA023 |  | Acanthostrongylophora ingens | PV351299 | PV351571 | PV350300 | Taiwan | Central Indo-Pacific | Southern China | 23.53745 | 119.5456 | <30m | 5 | 5 | 2023-04-18 |
| e1100004667 | TWA036 |  | Petrosia (Petrosia) aff. elephantotus | PV351300 | PV351572 | PV350308 | Taiwan | Central Indo-Pacific | Southern China | 23.25597 | 119.5102 | <30m | 10 | 10 | 2023-04-21 |
| e1100004670 | CEL724 |  | Xestospongia testudinaria | PV351301 | PV351573 | PV350201 | Indonesia | Central Indo-Pacific | Sulawesi Sea/Makassar Strait | -5.12543 | 119.3437 | <30m | 10.1 | 10.1 | 2018-04-28 |
| e1100004671 | SUB053 |  | Neopetrosia ovata | PV351302 | PV351574 | PV350284 | Curacao | Tropical Atlantic | Southern Caribbean | 11.98713 | -68.6474 | >30m | 167 | 167 | 2018-08 |
| e1100004672 | SUB130 |  | Xestospongia muta | PV351303 | PV351575 | PV350292 | Curacao | Tropical Atlantic | Southern Caribbean | 12.06531 | -68.8602 | <30m | 28 | 28 | 2018-11 |
| e1100004673 | BDR008 |  | Neopetrosia eurystomata | PV351304 | PV351576 | PV350144 | Bonaire | Tropical Atlantic | Southern Caribbean | 12.1469 | -68.2821 | >30m | 108 | 108 | 2013-05-30 |
| e1100004674 | SCR063 |  | Xestospongia muta | PV351305 | PV351577 | PV350275 | Curacao | Tropical Atlantic | Southern Caribbean | 12.08456 | -68.8983 | <30m | 27 | 27 | 2015-11 |
| e1100004676 | DOM044 |  | Neopetrosia eurystomata |  | PV351578 | PV350202 | Dominica | Tropical Atlantic | Eastern Caribbean | 15.42367 | 61.34653 | >30m | 144 | 144 | 2016-03-17 |
| e1100004677 | TWA015 |  | Petrosia (Petrosia) aff. elephantotus | PV351306 | PV351579 | PV350293 | Taiwan | Central Indo-Pacific | Southern China | 23.53745 | 119.5456 | <30m | 5 | 5 | 2023-04-18 |
| e1100004678 | TWA025 |  | Petrosia (Petrosia) aff. elephantotus | PV351307 | PV351580 | PV350301 | Taiwan | Central Indo-Pacific | Southern China | 23.25597 | 119.5102 | <30m | 8 | 8 | 2023-04-21 |
| e1100004679 | TWA050 |  | Petrosia (Petrosia) aff. elephantotus | PV351308 | PV351581 | PV350309 | Taiwan | Central Indo-Pacific | Southern China | 23.24941 | 119.6142 | <30m | 8 | 8 | 2023-04-21 |
| e1100004681 | CEL442 |  | Petrosia (Petrosia) lignosa |  | PV351582 | PV350195 | Indonesia | Central Indo-Pacific | Sulawesi Sea/Makassar Strait | -4.70905 | 118.9646 | <30m | 20 |  | 2018-05-08 |
| e1100004683 | SUB059 |  | Xestospongia muta | PV351309 |  | PV350285 | Curacao | Tropical Atlantic | Southern Caribbean | 11.98713 | -68.6474 | <30m | 15 | 15 | 2018-08 |
| e1100004685 | BDR013 |  | Neopetrosia eurystomata |  | PV351583 | PV350145 | Bonaire | Tropical Atlantic | Southern Caribbean | 12.1469 | -68.2821 | >30m | 111 | 111 | 2013-05-30 |
| e1100004688 | DOM049 |  | Xestospongia muta | PV351310 | PV351584 | PV350203 | Dominica | Tropical Atlantic | Eastern Caribbean | 15.42367 | 61.34653 | >30m | 140 | 140 | 2016-03-17 |
| e1100004689 | TWA016 |  | Xestospongia testudinaria | PV351311 | PV351585 | PV350294 | Taiwan | Central Indo-Pacific | Southern China | 23.53745 | 119.5456 | <30m | 5 | 5 | 2023-04-18 |
| e1100004690 | TWA027 |  | Petrosia (Petrosia) aff. elephantotus | PV351312 | PV351586 | PV350302 | Taiwan | Central Indo-Pacific | Southern China | 23.25597 | 119.5102 | <30m | 10 | 10 | 2023-04-21 |
| e1100004691 | TWA051 |  | Acanthostrongylophora ingens | PV351313 | PV351587 | PV350310 | Taiwan | Central Indo-Pacific | Southern China | 23.24941 | 119.6142 | <30m | 7 | 7 | 2023-04-21 |
| e1100004693 | CEL448 |  | Petrosia (Petrosia) lignosa |  | PV351588 | PV350196 | Indonesia | Central Indo-Pacific | Sulawesi Sea/Makassar Strait | -4.70905 | 118.9646 | <30m | 20 |  | 2018-05-09 |
| e1100004695 | SUB069 |  | Xestospongia muta | PV351314 | PV351589 | PV350286 | Curacao | Tropical Atlantic | Southern Caribbean | 11.98713 | -68.6474 | <30m | 13 | 13 | 2018-08 |
| e1100004697 | BDR037 |  | Neopetrosia eurystomata | PV351315 | PV351590 | PV350146 | Bonaire | Tropical Atlantic | Southern Caribbean | 12.137 | -68.286 | >30m | 88 | 88 | 2013-05-31 |
| e1100004701 | TWA017 |  | Xestospongia testudinaria | PV351316 | PV351591 | PV350295 | Taiwan | Central Indo-Pacific | Southern China | 23.53745 | 119.5456 | <30m | 5 | 5 | 2023-04-18 |
| e1100004702 | TWA030 |  | Xestospongia vansoesti | PV351317 | PV351592 | PV350303 | Taiwan | Central Indo-Pacific | Southern China | 23.25597 | 119.5102 | <30m | 10 | 10 | 2023-04-21 |
| e1100004703 | TWA061 |  | Xestospongia testudinaria | PV351318 | PV351593 | PV350311 | Taiwan | Central Indo-Pacific | Southern China | 23.49014 | 119.5247 | <30m | 10 | 10 | 2023-04-23 |
| e1100034946 | MDB399 |  | Neopetrosia carbonaria | PV351319 | PV351594 | PV350231 | Martinique | Tropical Atlantic | Eastern Caribbean | 14.7577 | -60.8809 | <30m | 2 | 2 | 2016-09-22 |
| e1100034947 | CAO573 |  | Neopetrosia eurystomata | PV351320 | PV351595 | PV350164 | Curacao | Tropical Atlantic | Southern Caribbean | 12.08319 | -68.8973 | >30m | 102 | 102 | 2023-03-30 |
| e1100034958 | SUB001 |  | Xestospongia muta | PV351321 | PV351596 | PV350280 | Curacao | Tropical Atlantic | Southern Caribbean | 11.98713 | -68.6474 | >30m | 144 | 144 | 2018-08 |
| e1100034959 | CAO576 |  | Neopetrosia ovata | PV351322 | PV351597 | PV350165 | Curacao | Tropical Atlantic | Southern Caribbean | 12.08319 | -68.8973 | >30m | 188 | 188 | 2023-03-30 |
| e1100034970 | SUB002 |  | Neopetrosia ovata | PV351323 | PV351598 | PV350281 | Curacao | Tropical Atlantic | Southern Caribbean | 11.98713 | -68.6474 | >30m | 165 | 165 | 2018-08 |
| e1100034971 | CAO577 |  | Neopetrosia ovata | PV351324 | PV351599 | PV350166 | Curacao | Tropical Atlantic | Southern Caribbean | 12.08319 | -68.8973 | >30m | 189 | 189 | 2023-03-30 |
| e1100034983 | CAO578 |  | Neopetrosia eurystomata | PV351325 | PV351600 | PV350167 | Curacao | Tropical Atlantic | Southern Caribbean | 12.08319 | -68.8973 | >30m | 104 | 104 | 2023-03-30 |
| e1100034988 | CEL165 |  | Xestospongia vansoesti |  |  | PV350173 | Indonesia | Central Indo-Pacific | Sulawesi Sea/Makassar Strait | -5.0547 | 119.4265 | <30m | 6 | 6 | 2018-04-29 |
| e1100034995 | CAO589 |  | Petrosia (Petrosia) nova spec Curacao | PV351326 | PV351601 | PV350168 | Curacao | Tropical Atlantic | Southern Caribbean | 12.08319 | -68.8973 | >30m | 151 | 151 | 2023-03-30 |
| e1100035000 | CEL167 |  | Xestospongia vansoesti |  |  | PV350174 | Indonesia | Central Indo-Pacific | Sulawesi Sea/Makassar Strait | -5.0547 | 119.4265 | <30m | 6 | 6 | 2018-04-29 |
| e1100035006 | SUB042 |  | Xestospongia muta | PV351327 | PV351602 | PV350283 | Curacao | Tropical Atlantic | Southern Caribbean | 11.98713 | -68.6474 | >30m | 85 | 85 | 2018-08-29 |
| e1100035007 | CAO595 |  | Petrosia (Petrosia) nova spec Curacao | PV351328 | PV351603 | PV350169 | Curacao | Tropical Atlantic | Southern Caribbean | 12.08319 | -68.8973 | >30m | 153 | 153 | 2023-03-30 |
| e1100035012 | CEL170 |  | Xestospongia vansoesti |  |  | PV350175 | Indonesia | Central Indo-Pacific | Sulawesi Sea/Makassar Strait | -5.0547 | 119.4265 | <30m | 6 | 6 | 2018-04-29 |

**Table S2.** PCR reaction mixes and thermal cycling conditions used to amplify sponge DNA markers for molecular identification. Each reaction was performed in a final volume of 25 µl. *Thermal cycling included 30 cycles, excluding the initial denaturation and final extension steps.

|  | PCR mix | | Thermal Cycler conditions PCR1 | |
| --- | --- | --- | --- | --- |
|  | Chemical and concentration | Reaction (µl) | Temp. | Time |
| 28S rRNA | mQ water  ThermoFisher Phire HS II Buffer (5x) Qiagen MgCl_2_ (25 mM)  Promega BSA (10 mg/ml)  Qiagen Q-solution  Forward primer (10 pMol/µl)  Reverse primer (10 pMol/µl)  dNTP (2.5 mM)  ThermoFisher Phire HS II Taq  Template DNA (1:10 dilution) | 7.9  5  1  1  5  1.3  1.3  0.5  0.5  1.5 | 98°C  98°C  51°C  72°C  72°C | 0:00:30  0:00:10*  0:00:10*  0:00:15*  0:05:00 |
| 18S rRNA | mQ water  ThermoFisher Phire HS II Buffer (5x) Qiagen MgCl_2_ (25 mM)  Promega BSA (10 mg/ml)  Forward primer (10 pMol/µl)  Reverse primer (10 pMol/µl)  dNTP (2.5 mM)  ThermoFisher Phire HS II Taq  Template DNA (1:10 dilution) | 7.9  5  1  1  1.3  1.3  0.5  0.5  1.5 | 98°C  98°C  55°C  72°C  72°C | 0:00:30  0:00:10*  0:00:10*  0:00:30*  0:05:00 |
| COI | mQ water  ThermoFisher Phire HS II Buffer (5x) Qiagen MgCl_2_ (25 mM)  Promega BSA (10 mg/ml)  Forward primer (10 pMol/µl)  Reverse primer (10 pMol/µl)  dNTP (2.5 mM)  ThermoFisher Phire HS II Taq  Template DNA (1:10 dilution) | 7.9  5  1  1  1.3  1.3  0.5  0.5  1.5 | 98°C  98°C  53°C  72°C  72°C | 0:00:30  0:00:10*  0:00:10*  0:00:15*  0:05:00 |

**Table S3.** Results of the PERMANOVA test conducted with sponge species, collection depth and marine ecoregions as factors.

|  | D.f. | Sum of squares | R^2^ | F | *p* |
| --- | --- | --- | --- | --- | --- |
| Species | 18 | 36.57677 | 0.53482 | 15.7793 | 0.001 |
| Collection Depth | 1 | 0.381506 | 0.005578 | 2.962482 | 0.001 |
| Marine Ecoregion | 4 | 2.439412 | 0.035669 | 4.735654 | 0.001 |
| Residual | 143 | 18.4154 | 0.269267 | - | - |
| Total | 168 | 68.39081 | 1 | - | - |

**Table S4.** Results of the multivariate pairwise Adonis test comparing each individual sponge species. Insignificant *p*-values are highlighted in red.

| Pairwise comparisons | Sum of squares | F.Model | R^2^ | *p* value |
| --- | --- | --- | --- | --- |
| Acanthostrongylophora ingens vs Neopetrosia carbonaria | 3.34945519 | 28.14681278 | 0.623450717 | 0.001 |
| Acanthostrongylophora ingens vs Neopetrosia chaliniformis | 0.926314353 | 6.280189628 | 0.309671149 | 0.001 |
| Acanthostrongylophora ingens vs Neopetrosia eurystomata | 2.633854422 | 21.90590902 | 0.487817962 | 0.001 |
| Acanthostrongylophora ingens vs Neopetrosia ovata | 2.211372648 | 16.73053883 | 0.527269295 | 0.001 |
| Acanthostrongylophora ingens vs Neopetrosia proxima | 1.291529645 | 9.701387008 | 0.39274665 | 0.001 |
| Acanthostrongylophora ingens vs Neopetrosia rosariensis | 0.960342239 | 6.811637883 | 0.343820028 | 0.003 |
| Acanthostrongylophora ingens vs Petrosia (Petrosia) aff. elephantotus | 3.597648981 | 25.14355023 | 0.533340194 | 0.001 |
| Acanthostrongylophora ingens vs Petrosia (Petrosia) lignosa | 1.313384291 | 9.562234335 | 0.42381593 | 0.004 |
| Acanthostrongylophora ingens vs Petrosia (Petrosia) nigricans | 1.661047456 | 10.95244032 | 0.457257806 | 0.003 |
| Acanthostrongylophora ingens vs Petrosia (Petrosia) nova spec Curacao | 1.336146268 | 9.620242506 | 0.425293518 | 0.003 |
| Acanthostrongylophora ingens vs Petrosia (Petrosia) weinbergi | 3.733371399 | 25.35167368 | 0.559001942 | 0.001 |
| Acanthostrongylophora ingens vs Xestospongia mamillata | 1.030298794 | 6.5406119 | 0.334718889 | 0.002 |
| Acanthostrongylophora ingens vs Xestospongia muta | 3.102800229 | 18.06844988 | 0.340474424 | 0.001 |
| Acanthostrongylophora ingens vs Xestospongia viridenigra | 3.040929914 | 26.16144146 | 0.620506334 | 0.001 |
| Neopetrosia carbonaria vs Neopetrosia chaliniformis | 1.989081198 | 22.06600448 | 0.710294254 | 0.002 |
| Neopetrosia carbonaria vs Neopetrosia eurystomata | 3.681929225 | 43.84079194 | 0.708929989 | 0.001 |
| Neopetrosia carbonaria vs Neopetrosia ovata | 2.420984568 | 33.21200448 | 0.768582825 | 0.002 |
| Neopetrosia carbonaria vs Neopetrosia proxima | 2.414296335 | 32.48354877 | 0.764614768 | 0.002 |
| Neopetrosia carbonaria vs Neopetrosia rosariensis | 1.743137239 | 24.07968206 | 0.75062097 | 0.014 |
| Neopetrosia carbonaria vs Petrosia (Petrosia) aff. elephantotus | 3.279017075 | 29.42891336 | 0.63384885 | 0.001 |
| Neopetrosia carbonaria vs Petrosia (Petrosia) lignosa | 1.672770523 | 25.16026641 | 0.758747415 | 0.012 |
| Neopetrosia carbonaria vs Petrosia (Petrosia) nigricans | 1.686158711 | 18.79011513 | 0.701382396 | 0.01 |
| Neopetrosia carbonaria vs Petrosia (Petrosia) nova spec Curacao | 1.752588223 | 25.40586176 | 0.760521071 | 0.009 |
| Neopetrosia carbonaria vs Petrosia (Petrosia) weinbergi | 3.20260644 | 28.39899079 | 0.654369843 | 0.001 |
| Neopetrosia carbonaria vs Xestospongia mamillata | 1.583935224 | 15.95674746 | 0.666064852 | 0.01 |
| Neopetrosia carbonaria vs Xestospongia muta | 4.002500186 | 25.24345889 | 0.456949282 | 0.001 |
| Neopetrosia carbonaria vs Xestospongia viridenigra | 1.794019331 | 32.55907255 | 0.747469371 | 0.001 |
| Neopetrosia chaliniformis vs Neopetrosia ovata | 1.506250653 | 13.6767105 | 0.661454853 | 0.007 |
| Neopetrosia chaliniformis vs Petrosia (Petrosia) lignosa | 1.053110098 | 9.175736227 | 0.647284633 | 0.023 |
| Neopetrosia chaliniformis vs Xestospongia mamillata | 0.798111785 | 4.772862872 | 0.488379192 | 0.026 |
| Neopetrosia chaliniformis vs Xestospongia viridenigra | 1.875058603 | 23.14587475 | 0.743144154 | 0.007 |
| Neopetrosia eurystomata vs Neopetrosia chaliniformis | 1.508430869 | 14.56302303 | 0.4926094 | 0.001 |
| Neopetrosia eurystomata vs Neopetrosia ovata | 2.219001861 | 24.13007106 | 0.601296495 | 0.001 |
| Neopetrosia eurystomata vs Petrosia (Petrosia) lignosa | 1.517040411 | 16.66699828 | 0.543483197 | 0.004 |
| Neopetrosia eurystomata vs Xestospongia mamillata | 1.222024858 | 11.13443421 | 0.44299522 | 0.004 |
| Neopetrosia eurystomata vs Xestospongia viridenigra | 3.346172073 | 42.18333394 | 0.712756973 | 0.001 |
| Neopetrosia proxima vs Neopetrosia chaliniformis | 0.989556004 | 8.821642776 | 0.557568067 | 0.007 |
| Neopetrosia proxima vs Neopetrosia eurystomata | 0.692826192 | 7.461536361 | 0.318032726 | 0.001 |
| Neopetrosia proxima vs Neopetrosia ovata | 1.644127802 | 18.71295405 | 0.700519831 | 0.014 |
| Neopetrosia proxima vs Petrosia (Petrosia) aff. elephantotus | 2.413385565 | 19.37837527 | 0.563679206 | 0.001 |
| Neopetrosia proxima vs Petrosia (Petrosia) lignosa | 1.177517366 | 13.9678595 | 0.699517116 | 0.02 |
| Neopetrosia proxima vs Petrosia (Petrosia) nigricans | 1.413825416 | 12.2616773 | 0.671443104 | 0.019 |
| Neopetrosia proxima vs Petrosia (Petrosia) nova spec Curacao | 1.032050172 | 11.77683358 | 0.662482074 | 0.021 |
| Neopetrosia proxima vs Xestospongia mamillata | 0.9300405 | 7.265478256 | 0.547698177 | 0.019 |
| Neopetrosia proxima vs Xestospongia muta | 1.208880596 | 7.155237458 | 0.203532616 | 0.001 |
| Neopetrosia proxima vs Xestospongia viridenigra | 2.276591427 | 35.32401267 | 0.796949792 | 0.004 |
| Neopetrosia rosariensis vs Neopetrosia chaliniformis | 0.770551988 | 6.203103082 | 0.553695082 | 0.032 |
| Neopetrosia rosariensis vs Neopetrosia eurystomata | 1.165667781 | 12.34878568 | 0.468666216 | 0.002 |
| Neopetrosia rosariensis vs Neopetrosia ovata | 1.303379731 | 14.51506847 | 0.707532051 | 0.011 |
| Neopetrosia rosariensis vs Neopetrosia proxima | 0.774301932 | 8.400230421 | 0.583340001 | 0.017 |
| Neopetrosia rosariensis vs Petrosia (Petrosia) aff. elephantotus | 1.605147104 | 12.24592342 | 0.485065379 | 0.003 |
| Neopetrosia rosariensis vs Petrosia (Petrosia) lignosa | 1.017025962 | 11.90555729 | 0.74851557 | 0.1 |
| Neopetrosia rosariensis vs Petrosia (Petrosia) nigricans | 1.173962252 | 8.898493451 | 0.689886263 | 0.1 |
| Neopetrosia rosariensis vs Petrosia (Petrosia) nova spec Curacao | 0.96662878 | 10.69013736 | 0.727708469 | 0.1 |
| Neopetrosia rosariensis vs Petrosia (Petrosia) weinbergi | 1.730795916 | 12.68067989 | 0.53548631 | 0.003 |
| Neopetrosia rosariensis vs Xestospongia mamillata | 0.856121947 | 5.67028884 | 0.58636189 | 0.1 |
| Neopetrosia rosariensis vs Xestospongia muta | 0.969590482 | 5.520498853 | 0.175139958 | 0.002 |
| Neopetrosia rosariensis vs Xestospongia viridenigra | 1.676156907 | 28.20958933 | 0.801190524 | 0.009 |
| Petrosia (Petrosia) aff. elephantotus vs Neopetrosia chaliniformis | 1.996610172 | 14.43720427 | 0.507687188 | 0.002 |
| Petrosia (Petrosia) aff. elephantotus vs Neopetrosia eurystomata | 4.245265581 | 37.03325485 | 0.616879011 | 0.001 |
| Petrosia (Petrosia) aff. elephantotus vs Neopetrosia ovata | 2.51245454 | 20.3293404 | 0.575423718 | 0.001 |
| Petrosia (Petrosia) aff. elephantotus vs Petrosia (Petrosia) lignosa | 1.568168978 | 12.30498918 | 0.486267316 | 0.001 |
| Petrosia (Petrosia) aff. elephantotus vs Petrosia (Petrosia) nova spec Curacao | 1.78514823 | 13.84055044 | 0.515658219 | 0.002 |
| Petrosia (Petrosia) aff. elephantotus vs Xestospongia mamillata | 1.389196054 | 9.411015561 | 0.419928117 | 0.003 |
| Petrosia (Petrosia) aff. elephantotus vs Xestospongia muta | 4.82265133 | 28.69872467 | 0.45053845 | 0.001 |
| Petrosia (Petrosia) aff. elephantotus vs Xestospongia viridenigra | 2.942016883 | 27.19415174 | 0.629579483 | 0.001 |
| Petrosia (Petrosia) elephantotus vs Acanthostrongylophora ingens | 1.664866621 | 13.06783154 | 0.501301058 | 0.002 |
| Petrosia (Petrosia) elephantotus vs Neopetrosia carbonaria | 1.797853523 | 35.73052677 | 0.817061431 | 0.006 |
| Petrosia (Petrosia) elephantotus vs Neopetrosia chaliniformis | 1.317730674 | 14.82211169 | 0.747756441 | 0.029 |
| Petrosia (Petrosia) elephantotus vs Neopetrosia eurystomata | 1.858462633 | 22.72459579 | 0.618784096 | 0.004 |
| Petrosia (Petrosia) elephantotus vs Neopetrosia ovata | 1.505757937 | 24.94471889 | 0.806105849 | 0.021 |
| Petrosia (Petrosia) elephantotus vs Neopetrosia proxima | 1.398745581 | 22.29249722 | 0.787929642 | 0.015 |
| Petrosia (Petrosia) elephantotus vs Neopetrosia rosariensis | 1.159364927 | 21.83802087 | 0.845189381 | 0.1 |
| Petrosia (Petrosia) elephantotus vs Petrosia (Petrosia) aff. elephantotus | 1.181566955 | 10.0565368 | 0.436168575 | 0.004 |
| Petrosia (Petrosia) elephantotus vs Petrosia (Petrosia) lignosa | 1.115460556 | 27.02328519 | 0.87106459 | 0.1 |
| Petrosia (Petrosia) elephantotus vs Petrosia (Petrosia) nigricans | 1.244267775 | 14.17460384 | 0.779912672 | 0.1 |
| Petrosia (Petrosia) elephantotus vs Petrosia (Petrosia) nova spec Curacao | 1.284838949 | 27.76483348 | 0.87407458 | 0.1 |
| Petrosia (Petrosia) elephantotus vs Petrosia (Petrosia) weinbergi | 1.911309963 | 15.86973386 | 0.590617456 | 0.006 |
| Petrosia (Petrosia) elephantotus vs Xestospongia mamillata | 1.074050556 | 10.05315943 | 0.715366497 | 0.1 |
| Petrosia (Petrosia) elephantotus vs Xestospongia muta | 1.844716427 | 10.92564626 | 0.29588233 | 0.002 |
| Petrosia (Petrosia) elephantotus vs Xestospongia nova spec Lanyu | 2.036771346 | 74.91502283 | 0.882235208 | 0.007 |
| Petrosia (Petrosia) elephantotus vs Xestospongia viridenigra | 1.673540892 | 48.94641377 | 0.874880273 | 0.011 |
| Petrosia (Petrosia) lignosa vs Neopetrosia ovata | 1.376094327 | 16.79790579 | 0.736817932 | 0.017 |
| Petrosia (Petrosia) nigricans vs Neopetrosia chaliniformis | 1.258919003 | 8.283769759 | 0.623600823 | 0.027 |
| Petrosia (Petrosia) nigricans vs Neopetrosia eurystomata | 1.801214195 | 17.26832714 | 0.552262584 | 0.004 |
| Petrosia (Petrosia) nigricans vs Neopetrosia ovata | 1.440174282 | 12.75358719 | 0.68006121 | 0.016 |
| Petrosia (Petrosia) nigricans vs Petrosia (Petrosia) aff. elephantotus | 1.669566627 | 11.77820133 | 0.475345291 | 0.001 |
| Petrosia (Petrosia) nigricans vs Petrosia (Petrosia) lignosa | 1.175484588 | 9.786190037 | 0.709854573 | 0.1 |
| Petrosia (Petrosia) nigricans vs Petrosia (Petrosia) nova spec Curacao | 1.169414124 | 9.346737622 | 0.700301294 | 0.1 |
| Petrosia (Petrosia) nigricans vs Xestospongia mamillata | 1.049939685 | 5.654686104 | 0.585693418 | 0.1 |
| Petrosia (Petrosia) nigricans vs Xestospongia muta | 1.796186185 | 9.925224819 | 0.276274536 | 0.001 |
| Petrosia (Petrosia) nigricans vs Xestospongia viridenigra | 1.618172619 | 20.42061827 | 0.744717645 | 0.014 |
| Petrosia (Petrosia) nova spec Curacao vs Neopetrosia chaliniformis | 1.000047704 | 8.420065204 | 0.627423569 | 0.031 |
| Petrosia (Petrosia) nova spec Curacao vs Neopetrosia eurystomata | 1.231428651 | 13.32014142 | 0.487557558 | 0.003 |
| Petrosia (Petrosia) nova spec Curacao vs Neopetrosia ovata | 1.164184859 | 13.65571156 | 0.694745215 | 0.01 |
| Petrosia (Petrosia) nova spec Curacao vs Petrosia (Petrosia) lignosa | 1.101686737 | 14.01441754 | 0.77795563 | 0.1 |
| Petrosia (Petrosia) nova spec Curacao vs Xestospongia mamillata | 0.901590701 | 6.253650091 | 0.609895016 | 0.1 |
| Petrosia (Petrosia) nova spec Curacao vs Xestospongia viridenigra | 1.686034151 | 30.36556516 | 0.812661739 | 0.014 |
| Petrosia (Petrosia) nova spec Lanyu vs Acanthostrongylophora ingens | 2.907673102 | 22.67729382 | 0.557492687 | 0.001 |
| Petrosia (Petrosia) nova spec Lanyu vs Neopetrosia carbonaria | 3.087040386 | 38.06592217 | 0.745427098 | 0.001 |
| Petrosia (Petrosia) nova spec Lanyu vs Neopetrosia chaliniformis | 1.734474933 | 15.82199528 | 0.612733257 | 0.002 |
| Petrosia (Petrosia) nova spec Lanyu vs Neopetrosia eurystomata | 3.285594626 | 34.74544881 | 0.646481694 | 0.001 |
| Petrosia (Petrosia) nova spec Lanyu vs Neopetrosia ovata | 2.360654693 | 25.61094123 | 0.699543371 | 0.001 |
| Petrosia (Petrosia) nova spec Lanyu vs Neopetrosia proxima | 2.033776489 | 21.75799926 | 0.664204156 | 0.001 |
| Petrosia (Petrosia) nova spec Lanyu vs Neopetrosia rosariensis | 1.548332906 | 16.12688422 | 0.641817906 | 0.004 |
| Petrosia (Petrosia) nova spec Lanyu vs Petrosia (Petrosia) aff. elephantotus | 3.367024432 | 27.8122275 | 0.607091797 | 0.001 |
| Petrosia (Petrosia) nova spec Lanyu vs Petrosia (Petrosia) elephantotus | 1.808311015 | 23.67250511 | 0.724539028 | 0.006 |
| Petrosia (Petrosia) nova spec Lanyu vs Petrosia (Petrosia) lignosa | 1.575583388 | 17.35991472 | 0.658572492 | 0.013 |
| Petrosia (Petrosia) nova spec Lanyu vs Petrosia (Petrosia) nigricans | 1.671200202 | 14.99800296 | 0.624968794 | 0.006 |
| Petrosia (Petrosia) nova spec Lanyu vs Petrosia (Petrosia) nova spec Curacao | 1.615550322 | 17.37502198 | 0.658768057 | 0.003 |
| Petrosia (Petrosia) nova spec Lanyu vs Petrosia (Petrosia) weinbergi | 3.282640687 | 26.57283242 | 0.624173467 | 0.001 |
| Petrosia (Petrosia) nova spec Lanyu vs Xestospongia mamillata | 1.423150196 | 11.86973844 | 0.568753579 | 0.004 |
| Petrosia (Petrosia) nova spec Lanyu vs Xestospongia muta | 3.310130344 | 20.35330648 | 0.396338773 | 0.001 |
| Petrosia (Petrosia) nova spec Lanyu vs Xestospongia nova spec Lanyu | 3.486442103 | 56.62227588 | 0.790567951 | 0.001 |
| Petrosia (Petrosia) nova spec Lanyu vs Xestospongia viridenigra | 2.868827193 | 38.63451078 | 0.763007486 | 0.001 |
| Petrosia (Petrosia) weinbergi vs Neopetrosia chaliniformis | 2.013921113 | 13.94073493 | 0.537407092 | 0.001 |
| Petrosia (Petrosia) weinbergi vs Neopetrosia eurystomata | 4.171760837 | 35.99316461 | 0.631534761 | 0.001 |
| Petrosia (Petrosia) weinbergi vs Neopetrosia ovata | 2.510612593 | 19.76591847 | 0.603246281 | 0.001 |
| Petrosia (Petrosia) weinbergi vs Neopetrosia proxima | 2.499380984 | 19.50868464 | 0.600106859 | 0.001 |
| Petrosia (Petrosia) weinbergi vs Petrosia (Petrosia) aff. elephantotus | 3.819700341 | 27.12428589 | 0.575590386 | 0.001 |
| Petrosia (Petrosia) weinbergi vs Petrosia (Petrosia) lignosa | 1.816584195 | 13.74163052 | 0.555405211 | 0.002 |
| Petrosia (Petrosia) weinbergi vs Petrosia (Petrosia) nigricans | 1.631221704 | 10.9400045 | 0.498632737 | 0.007 |
| Petrosia (Petrosia) weinbergi vs Petrosia (Petrosia) nova spec Curacao | 1.74624305 | 13.03038691 | 0.542246238 | 0.005 |
| Petrosia (Petrosia) weinbergi vs Xestospongia mamillata | 1.539617668 | 9.867101941 | 0.472854447 | 0.006 |
| Petrosia (Petrosia) weinbergi vs Xestospongia muta | 4.639814225 | 26.96163419 | 0.449648088 | 0.001 |
| Petrosia (Petrosia) weinbergi vs Xestospongia viridenigra | 2.957604601 | 27.09172564 | 0.659298806 | 0.001 |
| Petrosia (Strongylophora) corticata vs Acanthostrongylophora ingens | 2.83222374 | 21.8087886 | 0.576817968 | 0.001 |
| Petrosia (Strongylophora) corticata vs Neopetrosia carbonaria | 2.675388767 | 35.70777344 | 0.764493163 | 0.001 |
| Petrosia (Strongylophora) corticata vs Neopetrosia chaliniformis | 1.787561627 | 16.51042975 | 0.673608334 | 0.011 |
| Petrosia (Strongylophora) corticata vs Neopetrosia eurystomata | 3.125528108 | 33.91714388 | 0.666124242 | 0.001 |
| Petrosia (Strongylophora) corticata vs Neopetrosia ovata | 2.125396195 | 24.40438764 | 0.730574316 | 0.003 |
| Petrosia (Strongylophora) corticata vs Neopetrosia proxima | 2.071998533 | 23.36534031 | 0.721924753 | 0.004 |
| Petrosia (Strongylophora) corticata vs Neopetrosia rosariensis | 1.544502039 | 17.05310292 | 0.708977257 | 0.017 |
| Petrosia (Strongylophora) corticata vs Petrosia (Petrosia) aff. elephantotus | 2.875950448 | 23.60921977 | 0.596053644 | 0.001 |
| Petrosia (Strongylophora) corticata vs Petrosia (Petrosia) elephantotus | 1.696712325 | 25.96606546 | 0.787660435 | 0.013 |
| Petrosia (Strongylophora) corticata vs Petrosia (Petrosia) lignosa | 1.551461287 | 18.50928548 | 0.725590119 | 0.014 |
| Petrosia (Strongylophora) corticata vs Petrosia (Petrosia) nigricans | 1.543924068 | 13.98554648 | 0.666437088 | 0.016 |
| Petrosia (Strongylophora) corticata vs Petrosia (Petrosia) nova spec Curacao | 1.570972791 | 18.1245093 | 0.721387593 | 0.012 |
| Petrosia (Strongylophora) corticata vs Petrosia (Petrosia) nova spec Lanyu | 2.592453687 | 28.04846343 | 0.700363036 | 0.001 |
| Petrosia (Strongylophora) corticata vs Petrosia (Petrosia) weinbergi | 2.80951778 | 22.52188589 | 0.616668207 | 0.001 |
| Petrosia (Strongylophora) corticata vs Xestospongia mamillata | 1.432555296 | 11.81165555 | 0.627890274 | 0.015 |
| Petrosia (Strongylophora) corticata vs Xestospongia muta | 3.154064852 | 19.01008123 | 0.395960197 | 0.001 |
| Petrosia (Strongylophora) corticata vs Xestospongia nova spec Lanyu | 3.000422793 | 56.24347891 | 0.812256689 | 0.001 |
| Petrosia (Strongylophora) corticata vs Xestospongia vansoesti | 1.948474569 | 7.337237182 | 0.360778463 | 0.002 |
| Petrosia (Strongylophora) corticata vs Xestospongia viridenigra | 2.521172249 | 38.14335292 | 0.792287005 | 0.001 |
| Xestospongia mamillata vs Neopetrosia ovata | 1.247902618 | 9.933408583 | 0.623432741 | 0.02 |
| Xestospongia mamillata vs Petrosia (Petrosia) lignosa | 0.996950079 | 7.163422993 | 0.641686963 | 0.1 |
| Xestospongia mamillata vs Xestospongia viridenigra | 1.524769175 | 16.91725028 | 0.707324215 | 0.02 |
| Xestospongia muta vs Neopetrosia chaliniformis | 1.476144485 | 8.305636531 | 0.23524959 | 0.001 |
| Xestospongia muta vs Neopetrosia eurystomata | 2.92630425 | 19.15716212 | 0.347319575 | 0.001 |
| Xestospongia muta vs Neopetrosia ovata | 2.514783599 | 14.92983331 | 0.347772916 | 0.001 |
| Xestospongia muta vs Petrosia (Petrosia) lignosa | 1.586187745 | 9.125597174 | 0.259799061 | 0.002 |
| Xestospongia muta vs Petrosia (Petrosia) nova spec Curacao | 1.47961293 | 8.47496374 | 0.245829519 | 0.003 |
| Xestospongia muta vs Xestospongia mamillata | 1.249484049 | 6.794238417 | 0.207177808 | 0.002 |
| Xestospongia muta vs Xestospongia viridenigra | 3.580271542 | 22.60331055 | 0.438020552 | 0.001 |
| Xestospongia nova spec Lanyu vs Acanthostrongylophora ingens | 3.628163093 | 36.42864848 | 0.657216971 | 0.001 |
| Xestospongia nova spec Lanyu vs Neopetrosia carbonaria | 3.292687428 | 72.18053498 | 0.837550324 | 0.001 |
| Xestospongia nova spec Lanyu vs Neopetrosia chaliniformis | 2.068974652 | 33.43809949 | 0.752464661 | 0.003 |
| Xestospongia nova spec Lanyu vs Neopetrosia eurystomata | 4.369809705 | 63.2821908 | 0.759852619 | 0.001 |
| Xestospongia nova spec Lanyu vs Neopetrosia ovata | 2.701273144 | 54.17986943 | 0.81867598 | 0.003 |
| Xestospongia nova spec Lanyu vs Neopetrosia proxima | 2.542931331 | 49.81434745 | 0.805870312 | 0.001 |
| Xestospongia nova spec Lanyu vs Neopetrosia rosariensis | 1.747019146 | 38.95557882 | 0.795733188 | 0.007 |
| Xestospongia nova spec Lanyu vs Petrosia (Petrosia) aff. elephantotus | 4.067694934 | 43.82524195 | 0.697573787 | 0.001 |
| Xestospongia nova spec Lanyu vs Petrosia (Petrosia) lignosa | 1.928199347 | 48.05860805 | 0.827760252 | 0.007 |
| Xestospongia nova spec Lanyu vs Petrosia (Petrosia) nigricans | 1.798740972 | 30.63077642 | 0.753881149 | 0.002 |
| Xestospongia nova spec Lanyu vs Petrosia (Petrosia) nova spec Curacao | 1.880077612 | 44.63512343 | 0.816967559 | 0.007 |
| Xestospongia nova spec Lanyu vs Petrosia (Petrosia) weinbergi | 3.730100023 | 40.62459334 | 0.704987072 | 0.001 |
| Xestospongia nova spec Lanyu vs Xestospongia mamillata | 1.72387003 | 25.98319525 | 0.722092495 | 0.004 |
| Xestospongia nova spec Lanyu vs Xestospongia muta | 4.86209183 | 33.6330059 | 0.512440432 | 0.001 |
| Xestospongia nova spec Lanyu vs Xestospongia viridenigra | 3.091781791 | 84.53775623 | 0.866718279 | 0.001 |
| Xestospongia testudinaria vs Acanthostrongylophora ingens | 2.037258183 | 11.04220475 | 0.269045116 | 0.001 |
| Xestospongia testudinaria vs Neopetrosia carbonaria | 3.592792404 | 20.97988954 | 0.456284035 | 0.001 |
| Xestospongia testudinaria vs Neopetrosia chaliniformis | 1.238503276 | 6.302505525 | 0.22268366 | 0.001 |
| Xestospongia testudinaria vs Neopetrosia eurystomata | 3.374967269 | 20.82630778 | 0.401848186 | 0.001 |
| Xestospongia testudinaria vs Neopetrosia ovata | 2.43295471 | 13.19489205 | 0.36455122 | 0.001 |
| Xestospongia testudinaria vs Neopetrosia proxima | 1.582351885 | 8.552915078 | 0.271065765 | 0.001 |
| Xestospongia testudinaria vs Neopetrosia rosariensis | 0.961108821 | 4.933516341 | 0.190237077 | 0.001 |
| Xestospongia testudinaria vs Petrosia (Petrosia) aff. elephantotus | 4.016000627 | 22.28594318 | 0.42623202 | 0.001 |
| Xestospongia testudinaria vs Petrosia (Petrosia) elephantotus | 1.646433464 | 8.83264449 | 0.296073132 | 0.002 |
| Xestospongia testudinaria vs Petrosia (Petrosia) lignosa | 1.32675057 | 6.889980422 | 0.247041422 | 0.001 |
| Xestospongia testudinaria vs Petrosia (Petrosia) nigricans | 1.681022339 | 8.345849203 | 0.284396241 | 0.002 |
| Xestospongia testudinaria vs Petrosia (Petrosia) nova spec Curacao | 1.376269023 | 7.111975246 | 0.25298739 | 0.002 |
| Xestospongia testudinaria vs Petrosia (Petrosia) nova spec Lanyu | 3.086093392 | 17.57227163 | 0.403290234 | 0.001 |
| Xestospongia testudinaria vs Petrosia (Petrosia) weinbergi | 4.222254263 | 22.71988243 | 0.447948247 | 0.001 |
| Xestospongia testudinaria vs Petrosia (Strongylophora) corticata | 2.883679742 | 15.96095255 | 0.399413716 | 0.001 |
| Xestospongia testudinaria vs Xestospongia mamillata | 1.105526464 | 5.391501996 | 0.20428932 | 0.002 |
| Xestospongia testudinaria vs Xestospongia muta | 3.30720643 | 17.19876517 | 0.285699634 | 0.001 |
| Xestospongia testudinaria vs Xestospongia nova spec Lanyu | 4.342255325 | 28.24690323 | 0.511284825 | 0.001 |
| Xestospongia testudinaria vs Xestospongia vansoesti | 2.576505637 | 10.06834999 | 0.271615812 | 0.001 |
| Xestospongia testudinaria vs Xestospongia viridenigra | 3.214971563 | 18.73691895 | 0.438424655 | 0.001 |
| Xestospongia vansoesti vs Acanthostrongylophora ingens | 2.421068896 | 9.890206739 | 0.342337693 | 0.001 |
| Xestospongia vansoesti vs Neopetrosia carbonaria | 2.273786164 | 9.369791466 | 0.400936032 | 0.001 |
| Xestospongia vansoesti vs Neopetrosia chaliniformis | 1.372199828 | 4.388630052 | 0.285186533 | 0.003 |
| Xestospongia vansoesti vs Neopetrosia eurystomata | 2.779450367 | 13.42788061 | 0.401697038 | 0.001 |
| Xestospongia vansoesti vs Neopetrosia ovata | 1.753657485 | 6.268557067 | 0.343133672 | 0.001 |
| Xestospongia vansoesti vs Neopetrosia proxima | 1.712873927 | 6.096824976 | 0.336900256 | 0.001 |
| Xestospongia vansoesti vs Neopetrosia rosariensis | 1.225135022 | 3.819917272 | 0.276406667 | 0.007 |
| Xestospongia vansoesti vs Petrosia (Petrosia) aff. elephantotus | 2.511338516 | 10.55120398 | 0.357048193 | 0.001 |
| Xestospongia vansoesti vs Petrosia (Petrosia) elephantotus | 1.38844652 | 4.581360551 | 0.314192941 | 0.01 |
| Xestospongia vansoesti vs Petrosia (Petrosia) lignosa | 1.279895375 | 4.050323509 | 0.288272616 | 0.005 |
| Xestospongia vansoesti vs Petrosia (Petrosia) nigricans | 1.153716521 | 3.448049047 | 0.256397715 | 0.006 |
| Xestospongia vansoesti vs Petrosia (Petrosia) nova spec Curacao | 1.245846835 | 3.917787992 | 0.281495019 | 0.003 |
| Xestospongia vansoesti vs Petrosia (Petrosia) nova spec Lanyu | 2.263523014 | 9.22037845 | 0.380686803 | 0.001 |
| Xestospongia vansoesti vs Petrosia (Petrosia) weinbergi | 2.264508269 | 8.91191172 | 0.343931078 | 0.001 |
| Xestospongia vansoesti vs Xestospongia mamillata | 1.01514128 | 2.966323399 | 0.228771357 | 0.013 |
| Xestospongia vansoesti vs Xestospongia muta | 2.87670506 | 12.46543697 | 0.280339918 | 0.001 |
| Xestospongia vansoesti vs Xestospongia nova spec Lanyu | 2.665687248 | 13.05609581 | 0.449341023 | 0.001 |
| Xestospongia vansoesti vs Xestospongia viridenigra | 2.106808779 | 8.468375611 | 0.394458145 | 0.001 |
| Xestospongia viridenigra vs Neopetrosia ovata | 2.293341628 | 36.48257923 | 0.80212204 | 0.004 |
| Xestospongia viridenigra vs Petrosia (Petrosia) lignosa | 1.584286228 | 30.0803156 | 0.811220593 | 0.014 |

**Table S5.** Results of the Kruskal-Wallis test comparing the Shannon, Chao1, and Pielou indices across different sponge species to assess whether alpha diversity differs significantly across species. A significant *p*-value (< 0.05) suggests that at least one species significantly differs in the respective alpha diversity index compared to the others.

|  | Chi-square | Df | *p* |
| --- | --- | --- | --- |
| Shannon | 123.2046 | 20 | < 0.001 |
| Chao1 | 101.4229 | 20 | < 0.001 |
| Pielou | 125.3356 | 20 | < 0.001 |

**Table S6.** Results of the pairwise Wilcoxon rank-sum test comparing the Shannon index between individual sponge species. The table provides *p*-values for each pairwise comparison, where values < 0.05 indicates significant differences. The final column provides a letter-based summary of similarities and differences, computed using ‘multcompLetters’ from the ‘multcompView’ package in R (Piepho, 2004).

|  | *Acanthostrongylophora ingens* | *Neopetrosia carbonaria* | *Neopetrosia chaliniformis* | *Neopetrosia eurystomata* | *Neopetrosia ovata* | *Neopetrosia proxima* | *Neopetrosia rosariensis* | *Petrosia (Petrosia)* aff. *elephantotus* | *Petrosia (Petrosia) elephantotus* | *Petrosia (Petrosia) lignosa* | *Petrosia (Petrosia) nigricans* | *Petrosia (Petrosia)* nova spec Curacao | *Petrosia (Petrosia)* nova spec Lanyu | *Petrosia (Petrosia) weinbergi* | *Petrosia (Strongylophora) corticata* | *Xestospongia mamillata* | *Xestospongia muta* | *Xestospongia* nova spec Lanyu | *Xestospongia testudinaria* | *Xestospongia vansoesti* | *Xestospongia viridenigra* |  |
| --- | --- | --- | --- | --- | --- | --- | --- | --- | --- | --- | --- | --- | --- | --- | --- | --- | --- | --- | --- | --- | --- | --- |
| *Acanthostrongylophora ingens* | 1 | 0.008 | 1 | 0.054 | 0.136 | 0.068 | 1 | 0.000 | 0.923 | 1 | 1 | 1 | 1 | 0.001 | 1 | 1 | 0.268 | 0.001 | 1 | 1 | 0.023 | ab |
| *Neopetrosia carbonaria* | 0.008 | 1 | 1 | 0.005 | 0.530 | 0.530 | 1 | 1 | 1 | 1 | 1 | 1 | 0.457 | 1 | 0.245 | 1 | 0.000 | 1 | 0.000 | 1 | 1 | cde |
| *Neopetrosia chaliniformis* | 1 | 1 | 1 | 0.706 | 1 | 1 | 1 | 1 | 1 | 1 | 1 | 1 | 1 | 0.420 | 1 | 1 | 0.477 | 0.587 | 1 | 1 | 1 | abcde |
| *Neopetrosia eurystomata* | 0.054 | 0.005 | 0.706 | 1 | 1 | 1 | 0.750 | 0.000 | 0.750 | 0.750 | 0.750 | 1 | 0.008 | 0.000 | 1 | 1 | 1 | 0.001 | 1 | 1 | 0.015 | a |
| *Neopetrosia ovata* | 0.136 | 0.530 | 1 | 1 | 1 | 1 | 1 | 0.068 | 1 | 1 | 1 | 1 | 0.326 | 0.140 | 1 | 1 | 1 | 0.210 | 0.656 | 1 | 0.909 | abcde |
| *Neopetrosia proxima* | 0.068 | 0.530 | 1 | 1 | 1 | 1 | 1 | 0.068 | 1 | 1 | 1 | 1 | 0.326 | 0.140 | 1 | 1 | 1 | 0.210 | 0.656 | 1 | 0.909 | abcde |
| *Neopetrosia rosariensis* | 1 | 1 | 1 | 0.750 | 1 | 1 | 1 | 1 | 1 | 1 | 1 | 1 | 1 | 1 | 1 | 1 | 1 | 1 | 1 | 1 | 1 | abcde |
| *Petrosia (Petrosia)* aff. *elephantotus* | 0.000 | 1 | 1 | 0.000 | 0.068 | 0.068 | 1 | 1 | 1 | 0.923 | 1 | 0.923 | 0.860 | 0.044 | 0.430 | 0.923 | 0.000 | 0.274 | 0.000 | 1 | 1 | cd |
| *Petrosia (Petrosia) elephantotus* | 0.923 | 1 | 1 | 0.750 | 1 | 1 | 1 | 1 | 1 | 1 | 1 | 1 | 1 | 1 | 1 | 1 | 0.128 | 1 | 0.474 | 1 | 1 | abcde |
| *Petrosia (Petrosia) lignosa* | 1 | 1 | 1 | 0.750 | 1 | 1 | 1 | 0.923 | 1 | 1 | 1 | 1 | 1 | 1 | 1 | 1 | 1 | 1 | 1 | 1 | 1 | abcde |
| *Petrosia (Petrosia) nigricans* | 1 | 1 | 1 | 0.750 | 1 | 1 | 1 | 1 | 1 | 1 | 1 | 1 | 1 | 1 | 1 | 1 | 0.513 | 1 | 0.949 | 1 | 1 | abcde |
| *Petrosia (Petrosia)* nova spec Curacao | 1 | 1 | 1 | 1 | 1 | 1 | 1 | 0.923 | 1 | 1 | 1 | 1 | 1 | 1 | 1 | 1 | 1 | 1 | 1 | 1 | 1 | abcde |
| *Petrosia (Petrosia)* nova spec Lanyu | 1 | 0.457 | 1 | 0.008 | 0.326 | 0.326 | 1 | 0.860 | 1 | 1 | 1 | 1 | 1 | 0.019 | 1 | 1 | 0.063 | 0.035 | 0.666 | 1 | 0.559 | bc |
| *Petrosia (Petrosia) weinbergi* | 0.001 | 1 | 0.420 | 0.000 | 0.140 | 0.140 | 1 | 0.044 | 1 | 1 | 1 | 1 | 0.019 | 1 | 0.052 | 1 | 0.000 | 1 | 0.000 | 0.055 | 1 | e |
| *Petrosia (Strongylophora) corticata* | 1 | 0.245 | 1 | 1 | 1 | 1 | 1 | 0.430 | 1 | 1 | 1 | 1 | 1 | 0.052 | 1 | 1 | 1 | 0.084 | 1 | 1 | 0.455 | abcde |
| *Xestospongia mamillata* | 1 | 1 | 1 | 1 | 1 | 1 | 1 | 0.923 | 1 | 1 | 1 | 1 | 1 | 1 | 1 | 1 | 1 | 1 | 1 | 1 | 1 | abcde |
| *Xestospongia muta* | 0.268 | 0.000 | 0.477 | 1 | 1 | 1 | 1 | 0.000 | 0.128 | 1 | 0.513 | 1 | 0.063 | 0.000 | 1 | 1 | 1 | 0.000 | 1 | 1 | 0.001 | ab |
| *Xestospongia* nova spec Lanyu | 0.001 | 1 | 0.587 | 0.001 | 0.210 | 0.210 | 1 | 0.274 | 1 | 1 | 1 | 1 | 0.035 | 1 | 0.084 | 1 | 0.000 | 1 | 0.000 | 0.164 | 1 | de |
| *Xestospongia testudinaria* | 1 | 0.000 | 1 | 1 | 0.656 | 0.656 | 1 | 0.000 | 0.474 | 1 | 0.949 | 1 | 0.666 | 0.000 | 1 | 1 | 1 | 0.000 | 1 | 1 | 0.002 | ab |
| *Xestospongia vansoesti* | 1 | 1 | 1 | 1 | 1 | 1 | 1 | 1 | 1 | 1 | 1 | 1 | 1 | 0.055 | 1 | 1 | 1 | 0.164 | 1 | 1 | 1 | abcde |
| *Xestospongia viridenigra* | 0.023 | 1 | 1 | 0.015 | 0.909 | 0.909 | 1 | 1 | 1 | 1 | 1 | 1 | 0.559 | 1 | 0.455 | 1 | 0.001 | 1 | 0.002 | 1 | 1 | cde |

## Supplementary references

Piepho, H.-P. (2004). An Algorithm for a Letter-Based Representation of All-Pairwise Comparisons. *Journal of Computational and Graphical Statistics*, *13*(2), 456–466. https://doi.org/10.1198/1061860043515

Stamatakis, A. (2014). RAxML version 8: A tool for phylogenetic analysis and post-analysis of large phylogenies. *Bioinformatics (Oxford, England)*, *30*(9), 1312–1313. https://doi.org/10.1093/bioinformatics/btu033
